# Consequences of Normalizing Transcriptomic and Genomic Libraries of Plant Genomes Using a Duplex-Specific Nuclease and Tetramethylammonium Chloride

**DOI:** 10.1371/journal.pone.0055913

**Published:** 2013-02-08

**Authors:** Marta Matvienko, Alexander Kozik, Lutz Froenicke, Dean Lavelle, Belinda Martineau, Bertrand Perroud, Richard Michelmore

**Affiliations:** 1 Genome Center, University of California Davis, Davis, California, United States of America; 2 Departments of Plant Sciences, Molecular and Cellular Biology, and Medical Microbiology and Immunology, University of California Davis, Davis, California, United States of America; Centro de Investigación y de Estudios Avanzados del IPN, Mexico

## Abstract

Several applications of high throughput genome and transcriptome sequencing would benefit from a reduction of the high-copy-number sequences in the libraries being sequenced and analyzed, particularly when applied to species with large genomes. We adapted and analyzed the consequences of a method that utilizes a thermostable duplex-specific nuclease for reducing the high-copy components in transcriptomic and genomic libraries prior to sequencing. This reduces the time, cost, and computational effort of obtaining informative transcriptomic and genomic sequence data for both fully sequenced and non-sequenced genomes. It also reduces contamination from organellar DNA in preparations of nuclear DNA. Hybridization in the presence of 3 M tetramethylammonium chloride (TMAC), which equalizes the rates of hybridization of GC and AT nucleotide pairs, reduced the bias against sequences with high GC content. Consequences of this method on the reduction of high-copy and enrichment of low-copy sequences are reported for Arabidopsis and lettuce.

## Introduction

The current generation of DNA sequencing technologies provides opportunities to generate massive amounts of sequence data for both transcriptomes and genomes. However, gene transcripts have large differences in representation in transcriptome libraries reflecting differential gene expression. Also, genomes of higher eukaryotes, especially those of animal and plant species, often contain highly repeated sequences of varying degrees of complexity and sequence divergence that are difficult to assemble and interfere with analyses of the low-copy genomic components. Some 35 to 50% of mammalian genomes [1], [2], [3] and more than 80% of some plant genomes [4], [5] are comprised of low complexity and highly repeated sequences. Conversely, the transcribed regions of many higher eukaryotic genomes comprise only a small part of the total genome and actual protein-coding regions comprise even less, e.g. approximately only 1.2% of the euchromatic human genome is protein encoding [6], [7]. The variable copy numbers of families of repeated elements that have diverged from one another over time [2], [8], [9] make assembly of shotgun-sequenced genomes problematic and inefficient [2], [10].

Several uses of sequence data, such as gene and single-nucleotide polymorphism (SNP) discovery as well as genotyping, would benefit from libraries with reduced representation of repeated sequences. In addition, protocols for genomic DNA isolation may result in a significant proportion of the DNA originating from mitochondria and, in the case of plants, chloroplasts. Several methods have been developed to enrich transcriptomic libraries for rare transcripts and genomic libraries for low-copy, predominantly genic, sequences. Historically, generation of cDNA libraries was used as one means of focusing on genic sequences; however, since levels of transcription vary by orders of magnitude, genes encoding rare transcripts have been difficult to identify. Also, cDNA libraries only comprise those transcripts being expressed in the tissue(s) used as the source(s) and, therefore, represent only a subset of genes. Because repetitive genomic DNA tends to be hypermethylated, some methods have employed methylation-sensitive restriction enzymes [11], [12] or bacterial host strains that preferentially restrict methylated DNA [13], [14]. However, genic regions also contain methylated DNA and so these methods inevitably result in partial representation of the gene-space. Other approaches to reduce complexity rely on selective capture using microarrays [15], [16] or amplification of specific sequences of the exome by PCR [7], [17], [18], [19]. These approaches are expensive and complex, however, as well as constrained by the sequences targeted for enrichment and potentially biased by differential amplification [7].

Additional methods for enriching for low-copy, protein-encoding sequences relative to repetitive sequences rely on the kinetics of nucleic acid renaturation. DNA fragments are heat-denatured and then allowed to renature under specific conditions of salt concentration and temperature. Complementary strands of repetitive sequences renature to form duplexes faster than low-copy sequences. Such DNA renaturation kinetics were the basis of C_o_t (initial concentration of nucleic acid x time) analysis that was originally used to estimate genome sizes as well as to examine the proportions and complexity of the low-copy and high-copy fractions of genomes [20]. It has been subsequently used to reduce the high-copy components of cDNA [21] and genomic libraries [22]. Physical separation of renatured and single-stranded fractions was achieved using chromatography over hydroxyapatite columns [21], [22], [23] or, for transcriptomes, immobilization of first strand cDNAs on solid beads [24] or biotinylation of the source RNA [25]. However, these methods are technically demanding and therefore not amenable to processing large numbers of samples. More recently, normalized cDNA [26] and genomic [27] libraries have been prepared by digesting renatured, double-stranded DNA/RNA hybrids or double-stranded DNA fragments, respectively, in solution using a duplex-specific nuclease from Kamchatka crab [28].

Here we describe our adaptation of these methods for normalizing transcriptomic and genomic libraries for the current generation of massively-parallel DNA sequencing technologies as well as the consequences of this protocol. The method utilizes the thermostable duplex-specific nuclease (DSN) from Kamchatka crab to eliminate renatured, high-copy sequences in solution as well as tetramethylammonium chloride (TMAC) to reduce bias due to differential hybridzation of GC- versus AT-rich sequences. We originally adapted a DSN normalization method for preparing cDNA libraries [26] for Sanger sequencing and subsequently for the RNA-Seq protocol from Illumina [29]. We then extended the protocol for Illumina sequencing of genomic DNA. We have demonstrated the efficacy and consequences of this normalization protocol with genomes of Arabidopsis and lettuce as well as the transcriptome of lettuce. This method is an efficient, inexpensive, simple procedure for reducing whole-genome redundancy or identifying rare transcripts as well as reducing contaminating organellar sequences that can also minimize bias related to GC content. The approach also reduces the computational effort required to obtain normalized transcriptomic and genomic data and facilitates studies of any organism, particularly those with large genome sizes.

## Results

### Experimental Overview

We have conducted a series of experiments examining the consequences of DSN treatment while generating sequencing libraries for a variety of studies over the past eight years. For each set of libraries we used what we considered to be the best protocol at that time in conjunction with the sequencing technology contemporaneously available. The protocol evolved as our understanding and the sequencing technology improved. Experiments were designed to address variables in the DSN protocol, particularly the efficacy of DSN treatment on the prevalence of different types of repeat sequences. As we analyzed the large amounts of data generated in each experiment, it progressively became apparent that the effects of DSN treatment were not as straightforward as we (and others) had assumed and subsequent experiments were designed accordingly. The results reported below for a subset of libraries that we made chronicle our understanding as it advanced. In some cases, datasets were analyzed retrospectively to gain greater understanding.

We had previously adapted the DSN normalization protocol (Evrogen, JSC, Moscow, Russia) for Sanger sequencing of cDNA libraries and used it, in conjunction with the SMART cDNA synthesis method (Clontech, Mountain View, CA, USA), to produce numerous normalized EST libraries from multiple plant species (http://compgenomics.ucdavis.edu). In 2007, we adapted the DSN protocol [26] to normalize cDNA libraries of lettuce for sequencing with early Illumina GAII chemistries. This demonstrated that RNA-Seq libraries generated from polyA RNA could be effectively normalized by DSN treatment. We then developed a protocol for reducing repeated sequences in libraries of genomic DNA. The prevalence of highly repeated sequences in genomic libraries of Arabidopsis was effectively reduced. However, analysis of Arabidopsis organellar DNA indicated potential bias against sequences with high GC content. We therefore explored the use of TMAC to reduce the bias due to GC content during renaturation of Arabidopsis genomic and organellar DNA. We subsequently generated extensive data comparing the effects of renaturation in TMAC and NaCl while generating libraries for sequencing the transcriptome and gene-space of lettuce.

### Normalization of Transcriptomic Libraries from Lettuce

**Figure 1:**
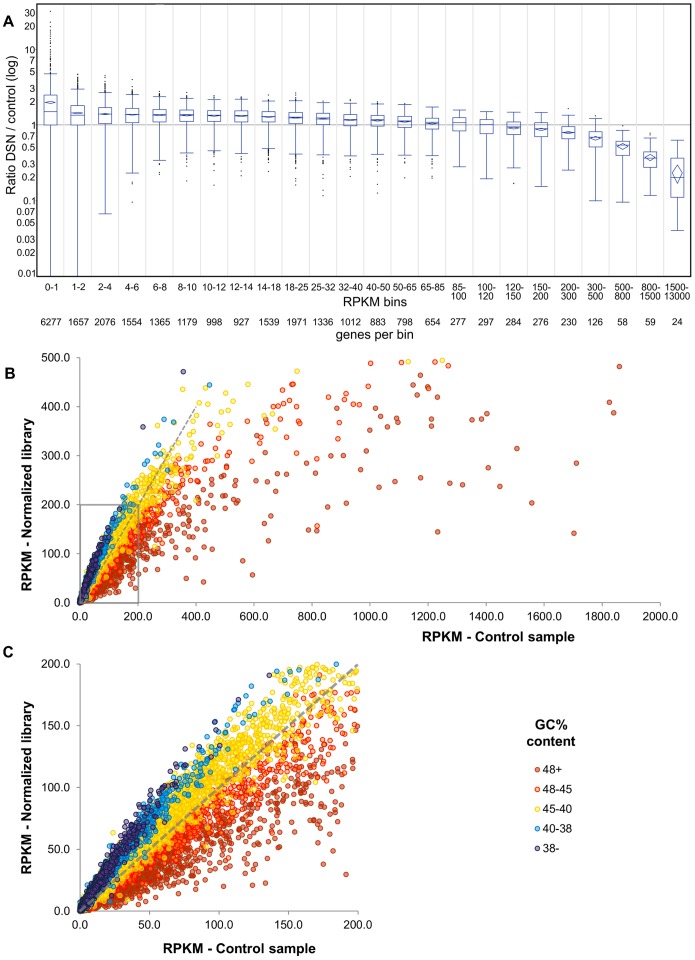
Plots depicting the effect of DSN treatment on RNA-Seq libraries of lettuce cultivar Valmaine. Abundance of each of 25,857 transcripts from a lettuce transcriptome assembly is given in reads per kilobase of exon per million mapped sequence reads (RPKM; [30]). The most highly abundant transcripts in the control library were the most depleted and *vice versa* for the less abundant transcripts. A) Boxplots of 24 bins based on transcript abundance of the RPKM ratio of the DSN normalized library over the control library. The whiskers encompass 1.5 of the interquartile range (IQR). The confidence diamonds indicate the average fold change when Student t-test p-value is less than 0.01 (see Table S1 for the actual p-values that range from 4×e^−195^ to 1×e^−4^ for the significant bins). A sequence with a ratio outside of the range covered by the whiskers is considered as an outlier and shown as a black dot. B) Scatter plot depicting the effect of DSN treatment on the abundance of reads representing each of 25,857 transcripts. Sequences that are neither reduced nor enhanced as a result of DSN treatment would align along the dotted blue lines. Color-coding indicates % GC content (see below). Sequences with high GC content (red dots) were more depleted than sequences with low GC content (blue and purple dots). C) Close-up of the section of the scatter plot in B (outlined in the blue rectangle), which highlights the effect of DSN treatment on transcripts represented in the libraries at lower abundance. A substantial number of the reads representing less abundant transcripts were enriched in the normalized library, particularly those with GC content of 45% or less.

We adapted the DSN protocol [26] to the RNA-Seq approach when sequencing using Illumina Genome Analyzers (IGA) became available. The RNA-Seq protocol was simpler and less expensive than the SMART approach and RNA fragmentation in the RNA-Seq approach produced less bias along the length of transcripts as compared to sequencing from the 3′ ends of transcripts [29], [30]. We compared a control (untreated) library prepared using leaf mRNA from lettuce cv. Valmaine to a normalized library generated from it by treatment with DSN (as per Evrogen JSC). Twelve million IGA reads (24 to 30 nt long) were produced from each library. These were analyzed using CLC Genomics Workbench (www.clcbio.com) to determine the abundance, expressed as reads per kilobase of exon per million mapped sequence reads (RPKM; [30]), of 25,857 transcripts from a lettuce transcriptome assembly (see below). The most highly abundant transcripts in the control library were the most depleted in the normalized library, as expected, while a substantial number of the less abundant transcripts were significantly enriched in the normalized library (Figure 1 and Table S1). The 83 most abundant transcripts from the control library were reduced 4.6 times overall in comparison with the normalized library (Table S1). The average level of enrichment of rare transcripts (those with RPKM values of 1 to 10 in the control library) in the normalized library as compared to the control library was 1.4 (Table S1).

**Figure 2:**
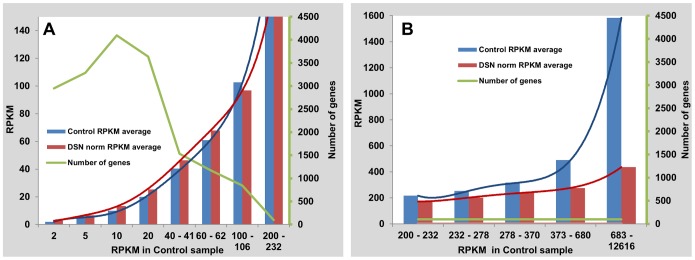
The consequences of DSN treatment on rare and abundant transcripts of lettuce. Reads in the control *L. sativa* cv. Valmaine RNA-Seq library were separated into bins based on their abundance expressed as RPKM (Table S1). The average RPKM values for 100 randomly selected genes in the control and the DSN-normalized libraries are shown as bars for each bin; the green line indicates the total number of genes in each bin. A) Less abundant transcripts (reads), representing the majority of genes expressed, are enriched in the DSN-treated library. B) The relatively few genes with highly abundant transcripts in the control library have substantially reduced abundance in the DSN-treated library.

To study both enrichment of rare transcripts and depletion of abundant transcripts in greater detail, sequencing reads were converted into RPKM and separated into bins based on their abundance in the control library. Transcriptome contigs with RPKM less than 2 were omitted from this analysis. The average RPKM for 100 genes in each bin was then determined both for the control and the DSN-treated libraries (Figure 2). For the relatively few genes with highly abundant transcripts in the control library, abundance in the DSN-treated library was substantially reduced (Figure 2A). Based on the control RNA-Seq library, however, the vast majority of lettuce genes were expressed at low levels and reads representing these genes were enriched in the DSN-treated library (Figure 2B). Sequences with RPKM of approximately 100 or higher were reduced and those with RPKMs of approximately 60 and lower were enriched as a result of DSN treatment.

### Reduction of Abundant Sequences in Genomic Libraries of Arabidopsis

We then extended the DSN protocol to genomic libraries. A time-course analysis was carried out on genomic libraries of Arabidopsis. A library of nuclear genomic DNA was prepared and denatured in a solution containing 0.5 M NaCl and sampled after renaturation at 68°C for 22, 46, 70 and 96 hours; these timepoints were chosen based on previous C_o_t analyses of genomic DNA [31], [32]. DSN was then added to digest the fraction that had reannealed. Approximately 3.8 million paired-end reads totalling 130 Mb were generated for each of the five libraries; this provided approximately 1× genome coverage for the control library and greater coverage for sequences retained in the DSN-treated libraries. BLAST analysis was used to determine the abundance levels of individual repeated sequences such as ribosomal RNAs and centromeric repeats as well as several transposon-related elements (http://plantrepeats.plantbiology.msu.edu/arabidopsis.html).

**Figure 3:**
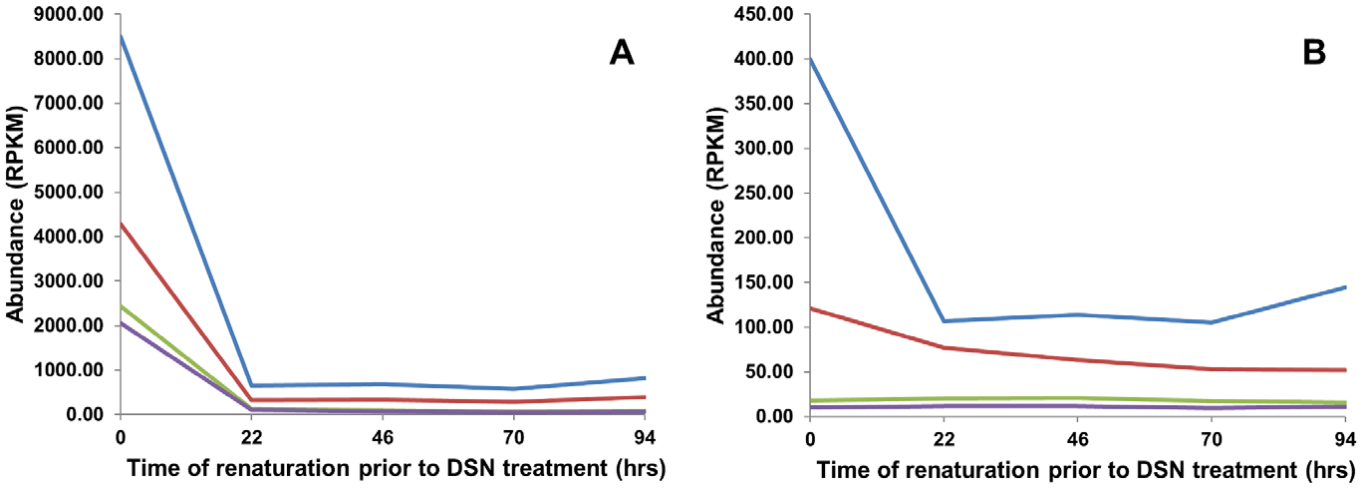
Effects of renaturation times on the abundance of repeated sequences in genomic libraries of Arabidopsis. A) Highly abundant, low complexity sequences were greatly decreased in samples that had been allowed to reanneal for 22 hrs or more prior to DSN treatment. Blue, 5S rRNA; red, repeat sequence AB073495.1 (GenBank ID: AB073495.1; http://www.ncbi.nlm.nih.gov/nuccore/AB073495.1); green, 18S rRNA; purple, 45S rDNA-like). B) Some moderately repeated Arabidopsis genomic sequences were also reduced in DSN-treated samples allowed to reanneal for 22 hrs or more while others were not. Blue, telomeric DNA; red, Athila gypsy-like (Class 1 RNA) retroelement; green, CACTA3 (Class 2 DNA) transposon; purple, copia-like (Class 1 RNA) retrotransposon.

The most highly abundant repeated sequences were greatly decreased in the DSN-treated libraries after 22 hrs of renaturation (Figure 3A). More moderately repeated sequences such as *Athila* retroelements were also reduced in the library which had been allowed to reanneal for 22 hours prior to DSN treatment and further reduction of copies of this *gypsy*-like Class 1 transposable element (TE) was also apparent in libraries allowed to reanneal for 46 and 70 hrs before DSN treatment (Figure 3B); further reduction of *Athila* sequences was not observed in the library allowed to reanneal for 94 hrs. As expected due to the low numbers of CACTA (approximately 100 total; [33]) and *Copia* transposable elements (8 or fewer/family; [34]), a member of the Class 2 (DNA) transposon CACTA3 superfamily and a Class 1 (RNA) *copia*-like retrotransposon were not reduced by DSN treatment, even after extended periods of renaturation (Figure 3B and data not shown).

**Figure 4:**
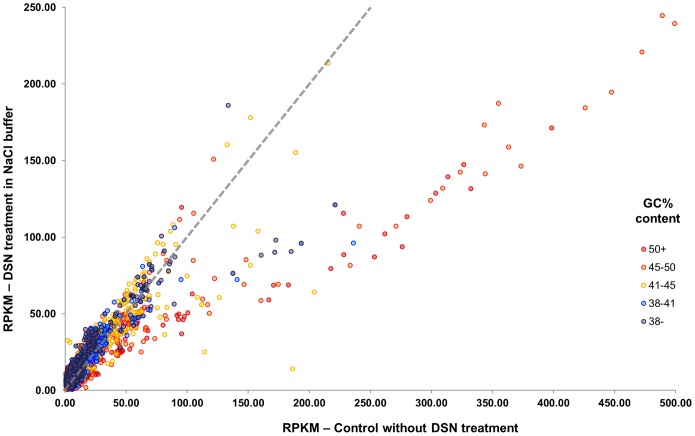
The majority of coding regions in Arabidopsis are not reduced as a result of DSN treatment. Abundance of more than 26,000 coding sequences compared between the control genomic library (x axis) and the library normalized with DSN after 22 hrs of renaturation (y axis) is expressed in RPKM. Sequences neither reduced nor enhanced as a result of DSN treatment align along the dotted gray line. The vast majority of coding sequences were at least as abundant in the normalized library as in the control library (for distribution and statistical significance, see Figure S1). Color-coding indicates % GC content. The few coding sequences that were reduced tended to have relatively high GC content (Student t-test P-value = 0.0452).

We then compared the library that had undergone DSN treatment after 22 hrs of renaturation to the control library for differential abundance of reads representing gene coding regions. This BLAST analysis revealed that most of more than 26,000 coding sequences were more abundant in the DSN-treated library than in the control library (Figure 4, Figure S1). We also examined the impact of DSN treatment on several large multigene families. MADS-box transcription factor family (108 copies; [35]), AP2/EREBP transcription factor family (138 copies; [36]), and ABC superfamily (136 members; [37]) members were enriched in the repeat-reduced libraries in aggregate by approximately 1.5, 1.2, and 1.2× respectively. However, representation of one multigene family (sequences related to At4G20530 that encode proteins of unknown function) was reduced as a result of DSN treatment; this family has 13 identical members and three nearly identical members [38], [39] and, therefore, would be predisposed to reduction.

### Analysis of Organellar and Genomic DNA of Arabidopsis Revealed a Bias Against Sequences with High GC Content

**Figure 5:**
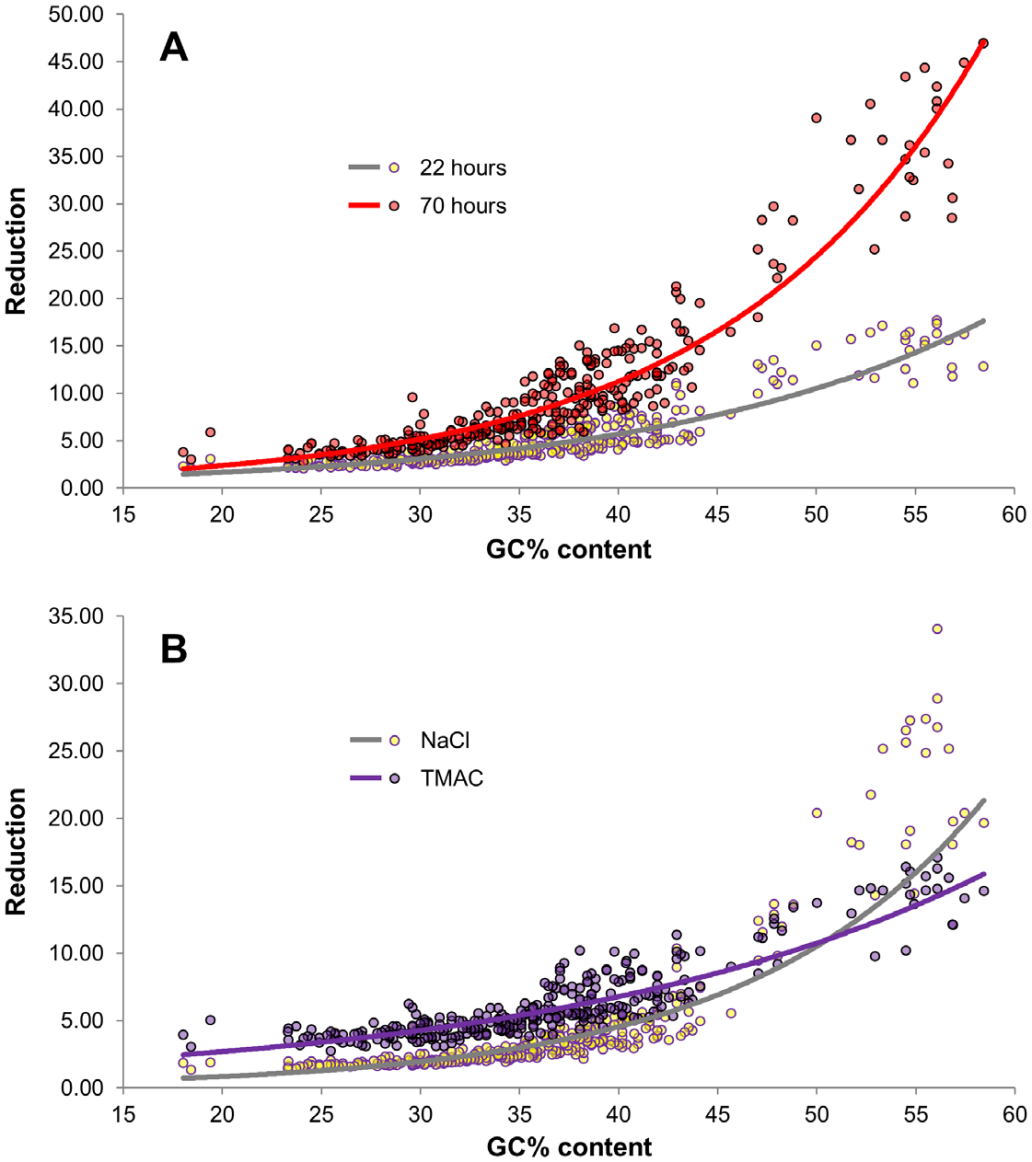
Hybridization in 3 M TMAC reduced GC bias associated with renaturation of Arabidiopsis chloroplast sequences. A) Renaturation was carried out in 0.5 M NaCl for 22 (yellow) or 70 (red) hrs prior to DSN treatment. The higher the GC content of the 500 nt fragment, the more it was reduced by DSN treatment. B) Renaturation was carried out for 22 hours in either 3 M TMAC (purple) or 0.5 M NaCl (yellow). Hybridization in 3 M TMAC reduced GC bias associated with renaturation followed by DSN treatment. Exponential curves that fit the data are indicated for each experiment. Statistical significances were assessed by two tail Student t-tests for each 5% GC content bin. All bins and conditions were statistically significant (see Table S2 for fold changes and P-values).

Many reads from the Arabidopsis genomic libraries were from chloroplast and mitochondrial genomes (ca. 16.8 and 2.4% of the total reads respectively in the control library); these were reduced by DSN treatment to acceptable levels compatible with the throughput of current sequencers (Table 1). After 22 hours of renaturation, the chloroplast and mitochondrial sequences had been reduced to 22% and 68% of their prevalence in the control libraries, respectively; however, after 70 hours of renaturation, chloroplast sequences had been further reduced to 12.4% and mitochondrial sequences were at their lowest level after 94 hours of renaturation. This progresive reduction over time might have been due to the AT-rich nature of some regions of the chloroplast and mitochondrial genomes. We therefore analyzed the extent of reduction of different regions of the chloroplast genome relative to GC content. The 154 kb Arabidopsis chloroplast genome [40] was divided into a contiguous tiling path of 500 nt fragments with 10 nt overlap and the GC content of each determined. The number of reads from each fragment after renaturation for 22 and 70 hours was determined using BLAST and the ratio of reads in the control and repeat-reduced libraries was plotted versus their GC content (Figure 5A, Table S2). The higher the GC content of a fragment, the more effectively the fragment was depleted (Figure 5A). This correlation was evident but not as great for the less abundant mitochondrial sequences (Figure S2). Re-examination of data from earlier experiments demonstrated a similar correlation between the extent of depletion and GC content (Figures 1B and 4, Table S2).

**Table 1:** Reduction of Arabidopsis organellar sequences renatured in 0.5 M NaCl for varying lengths of time and then treated with DSN.

	# Chloroplast Reads	# Mitochondrial Reads
0 hrs−DSN	637,508	89,756
22 hrs+DSN	141,235	61,172
46 hrs+DSN	109,516	46,243
70 hrs+DSN	79,305	34,111
94 hrs+DSN	83,457	29,819

### Repeat Reduction of Organellar and Genomic DNA of Arabidopsis Using Renaturation in Tetramethylammonium Chloride

We explored the use of tetramethylammonium chloride (TMAC) as a way to reduce bias resulting from differences in GC content during renaturation. Hybridization in the presence of 3 M TMAC has long been known to equalize the rates of reannealment of GC and AT nucleotide pairs [41], [42] and 3 M TMAC has been used to promote base composition-independent hybridization in the hybridization solutions and post-hybridization washes of Southern blot analyses [43] and lower concentrations of TMAC have been used to increase the thermostability of AT base pairs and thereby the specificity of PCR reactions (e.g. [44], [45]). However, TMAC had not previously been used to reduce repeated sequences in transcriptomic or genomic libraries. Therefore, to examine and potentially reduce bias in the repeat-reduction protocol, we compared the effects of renaturation of Arabidopsis genomic libraries in TMAC rather than NaCl prior to DSN treatment. Renaturations were carried out in either 3 M TMAC or 0.5 M NaCl for 22 hours at 68°C; samples were then treated with DSN or not and 3.8 million reads totalling approximately 128 Mb were generated for each of the four libraries.

Chloroplast fragments in each library were analyzed as above. Those with lower GC content were more reduced when renatured in TMAC, while fragments with higher GC content were reduced to a lesser extent compared to renaturation in NaCl; TMAC had the effect of flattening the curve (Figure 5B). Genomic repeated sequences with higher GC content, e.g. *Athila* (43% GC) and a 45S rDNA sequence (53.5% GC), were reduced to similar extents in 3 M TMAC and 0.5 M NaCl, while others with lower GC content, e.g. a centromeric repeat (37.8% GC), were more effectively reduced (by a factor of ∼5 for the centromeric repeat) when hybridization was carried out in 0.5 M NaCl (data not shown). We also compared the consequences of carrying out hybridizations in 3 M TMAC versus 0.5 M NaCl prior to DSN treatment on the abundance of approximately 20,000 genic regions. Little enrichment of single-copy genes was evident in the small Arabidopsis genome and the results were not statistically different (data not shown).

### Assembly of the Lettuce Transcriptome and the Effect of TMAC on the Content of Normalized RNA-Seq Libraries of Lettuce

We then examined the effect of TMAC on the GC content of normalized RNA-Seq libraries of lettuce. To assemble the lettuce transcriptome, we prepared two normalized RNA-Seq libraries from multiple tissues harvested from *L. sativa* cv. Salinas, one using 0.5 M NaCl and the other 3 M TMAC during renaturation for 5 hrs at 68°C prior to DSN treatment. A total of over 100 million 85 nt reads were generated (GenBank SRA SRR080725 and SRR085107). Ca. 80 million high quality reads were selected and assembled using Velvet [46], CLC Genomic Workbench (www.clcbio.com) and CAP3 [47]. Contigs of up to 15 Kb were assembled. The complete assembly had a total length of 53 Mb and provided 51,842 unigenes of 300 nt or longer with an average contig length of 1,020 nt and a median length of 736 nt. As evidenced by the presence of all 357 conserved orthologous sequences (COS) previously determined to be present in only one copy in many higher eukaryotes (A. Kozik, unpublished; http://compgenomics.ucdavis.edu/compositae_reference.php), this transcriptome assembly provides comprehensive coverage of most of the genes transcribed in lettuce. Approximately 67% of all the unigenes displayed strong similarity to known protein sequences in the Plant RefSeq database (RefSeq release 49, September 2011; [48]; http://www.ncbi.nlm.nih.gov/RefSeq/) and over 16,000 of them have complete uninterrupted open reading frames (ORFs).

**Figure 6:**
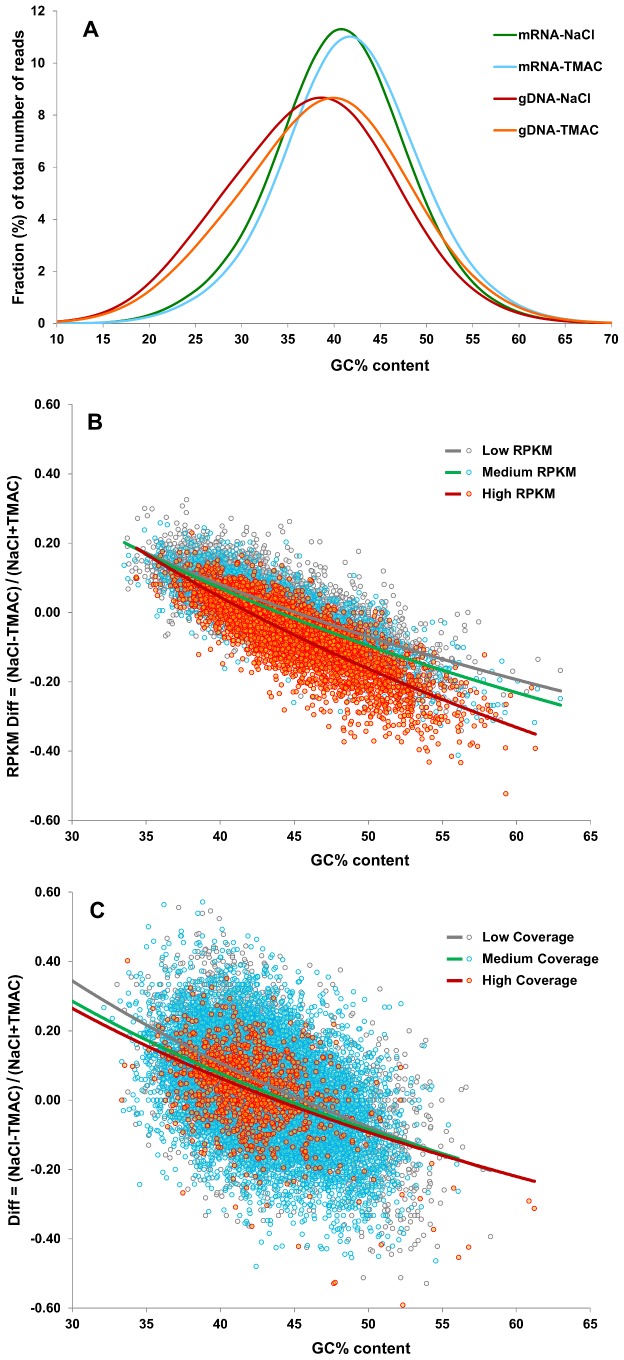
The effect of renaturation in 3 M TMAC versus 0.5 M NaCl on the GC composition of normalized transcriptomic and genomic libraries of lettuce. A) Twenty million reads in each RNA-Seq library (green: renatured in 0.5 M NaCl; blue: renatured in 3 M TMAC) and 10 million reads in each genomic library (red: renatured in 0.5 M NaCl; orange: renatured in 3 M TMAC) were categorized by % GC content and then the percentage of the total number of reads in each GC category was plotted against the % GC content of the category. The average GC content of reads in both types of library renatured in 3 M TMAC was approximately 1% greater than in the libraries renatured in 0.5 M NaCl (+1.1% for mRNA and +1.4% for gDNA). That shift is statistically significant based on a matched pairs difference analysis where the probability of being inferior to the Wilcoxon Signed Rank Test Statistic S is 0.9830. B) RNA-Seq reads from two libraries, one normalized using 3 M TMAC and the other 0.5 M NaCl, were separately mapped to a QC set of 25,857 uninterrupted ORFs identified in the lettuce transcriptome assembly. The differential abundance of reads representing each ORF in the libraries was then calculated by subtracting the RPKM for each gene in the TMAC-renatured library from the RPKM for that sequence in the NaCl-renatured library and the sum divided by the total RPKM for the gene in both libraries and these values plotted against the average GC content of that gene (Low coverage, <20 RPKM: gray dots. Medium coverage, 20 to 40 RPKM: blue dots. High coverage, 40 to 300 RPKM: red dots). Statistical significance was assessed by two tail Student t-tests for each RPKM bin. Differences between the hybridizations in NaCl and TMAC were small for transcripts present at moderate levels (Medium RPKM bin). However, NaCl was significantly more effective than TMAC both in reducing abundantly expressed transcripts (RPKM >40; average RPKM −16% with NaCl treatment; P-value = 2.6 e^−41^) and increasing the number of relatively rare transcripts (RPKM <20; average RPKM +5% with NaCl treatment; P-value = 9.8 e^−33^). The negative slopes of the regression lines (gray for low RPKM, green for medium RPKM, and red for high RPKM genes) indicate that genes with higher GC content tended to be represented at higher levels in the library renatured using 3 M TMAC as compared to 0.5 M NaCl and that, conversely, genes with lower GC content tended to be represented at higher levels in the library renatured using 0.5 M NaCl. This trend was more pronounced for genes with higher RPKM. C) Sixty million genomic reads from two libraries, one renatured using 3 M TMAC and the other 0.5 M NaCl, were separately mapped to a QC set of 25,857 uninterrupted ORFs identifed in the lettuce transcriptome assembly. The differential abundance of reads representing each gene in the two libraries was then calculated (as described in the text) and plotted against the average GC content of that gene (Low Coverage, <30 RPKM: gray circles. Medium coverage, 30 to 80 RPKM: blue circles. High coverage, >80 RPKM: red circles). The negative slopes of the regression lines (gray for low RPKM, green for medium RPKM, and red for high RPKM) indicate that genes with higher GC content tended to be represented at higher levels in the library normalized using 3 M TMAC as compared with 0.5 M NaCl and that genes with lower GC content tended to be represented at higher levels in the library renatured using 0.5 M NaCl. The low and medium RPKM bins were highly significantly different for both NaCl and TMAC treatments. The treatments were not significantly different for the high RPKM bin.

We utilized the lettuce RNA-Seq reads and transcriptome assembly to analyze the consequences of renaturation with TMAC in two ways. First, we categorized 20 million reads in each RNA-Seq library by GC content and then plotted the percentage of the total number of reads in each GC category against its GC composition (Figure 6A). The average GC content of the sequences was approximately 1% greater in the 3 M TMAC-renatured library than in the library renatured in 0.5 M NaCl. We also selected a set of 25,857 uninterrupted, partial or complete, ORFs based on homology of contigs to known proteins in the Plant RefSeq database. We mapped reads from the TMAC- and NaCl-renatured libraries back onto this QC set. The differential abundance of reads was calculated as above for Arabidopsis; this was then plotted against the average GC content for each gene (Figure 6B). The negative slope of the regression line indicates that the higher the GC content, the greater the enrichment in the TMAC-renatured library compared to the NaCl-renatured library.

### Normalization of Genomic Libraries, Gene-space Assembly of Lettuce

We used TMAC as well as NaCl while generating normalized genomic libraries for assembly of the gene-space of lettuce cv. Salinas. A total of 466 million reads (326 M paired and 140 M single) were assembled into 876,110 contigs using CLC Genomics Workbench (www.clcbio.com). The longest contig was 28 Kb; the average and median contig lengths were 1226 and 955 nt respectively. Total assembly length was 1.13 Gb (43% of the estimated length of the ∼2.7 Gb lettuce genome). More than 400 million reads mapped back to the assembly. This sequence assembly is available at NCBI GenBank under accession #AFSA00000000.1 (http://www.ncbi.nlm.nih.gov/nuccore/AFSA00000000.1). Alignments between the gene-space and transcriptome assemblies clearly identified the positions of the introns that usually matched the positions of introns in the corresponding genes in Arabidopsis (e.g. Figure S3).

We then compared the effect of renaturation in TMAC versus NaCl on the GC content of the genomic libraries. Ten million reads, each 50 nucleotides in length, from each of two genomic libraries, one renatured in 3 M TMAC and the other in 0.5 M NaCl for 22 hrs, were categorized by GC content and the percentage of the total number of reads in each GC category was plotted against their GC composition as was done for the RNA-Seq libraries. Again, GC content was approximately 1% greater in the genomic library in which renaturation had taken place in 3 M TMAC (Figure 6A). We also mapped the reads from the TMAC- and NaCl-renatured genomic libraries onto the same QC set of 25,857 uninterrupted ORFs that was utilized for the transcriptome analysis. The differential abundance of the reads in each library was then calculated and plotted against the average GC content for each gene (Figure 6C). The negative slope of the regression lines indicates that the higher the GC content of a gene, the more the sequences corresponding to that gene were enriched in the TMAC-normalized library than in the NaCl-normalized library.

### Effect of Normalization on Repeat Sequences in Gene-space Libraries of Lettuce

#### Simple sequence repeats

Centromeric-like repeat sequences [49], (CAA)n, (GAA)n, and the plant telomeric motif (TTTAGGG)n were identified as the most abundant simple sequence repeats in lettuce based on their prevalence in a non-normalized genomic library. To compare the abundance of these sequences in control and normalized genomic libraries, 28 million reads from each genomic library were mapped back to repeat sequences, each 126 nt long. Telomeric repeats, which were the most abundant simple repeated sequences in the control library, were depleted to the greatest extent in the normalized libraries; GAA repeats were also substantially reduced and CAA and centromeric repeats were both reduced by approximately a factor of two (Table 2). With the exception of the CAA repeat sequences, reduction was slightly more effective in 0.5 M NaCl than in 3 M TMAC (Table 2).

**Table 2:** Mapped reads from control and normalized genomic libraries.

Reference Sequence				Total number of mapped reads out of 28M [identity 80%]			Number of mapped reads per Kb out of 28M [identity 80%]			Fraction of mapped reads (%)			Reduction		Variability	
#	Description	Length	GC%	Control gDNA [DSN-]	NaCl buffer [DSN+]	TMAC buffer [DSN+]	Control gDNA [DSN-]	NaCl buffer [DSN+]	TMAC buffer [DSN+]	Control gDNA [DSN-]	NaCl buffer [DSN+]	TMAC buffer [DSN+]	NaCl	TMAC	Total # of variants	# of variants per Kb
**1**	**Telomere (TTTAGGG)n**	NA	42.9	16,068	38	132	NA	NA	NA	0.06	0.0001	0.0005	422.8	121.7	NA	NA
**2**	**(CAA)n**	NA	33.3	1,202	697	571	NA	NA	NA	0.004	0.002	0.002	1.7	2.1	NA	NA
**3**	**(GAA)n**	NA	33.3	337	77	103	NA	NA	NA	0.001	0.0003	0.0004	4.4	3.3	NA	NA
**4**	**centromeric tandem-9 DIMER**	340	37.1	8,200	3,881	5,595	24,118	11,415	16,456	0.03	0.01	0.02	2.1	1.5	91	268
**5**	**18S rRNA AH001680**	1,068	47.2	9,750	1,203	1,719	9,129	1,126	1,610	0.03	0.004	0.006	8.1	5.7	15	14
**6**	**28S rRNA AH001681**	444	57.9	2,393	114	281	5,390	257	633	0.01	0.0004	0.001	21.0	8.5	11	25
**7**	**Lsat_XCons_Pol_2012_05**	5,093	41.8	601,915	121,242	209,480	118,185	23,806	41,131	2.15	0.43	0.75	5.0	2.9	816	160
**8**	**Lsat_XCons_B449_2012_05**	5,888	35.5	827,744	134,225	198,431	140,582	22,796	33,701	2.96	0.48	0.71	6.2	4.2	757	129
**9**	**Lsat_MARPT_Cont_36**	209	38.3	13,938	2,543	3,822	66,689	12,167	18,287	0.05	0.01	0.01	5.5	3.6	24	115
**10**	**Lsat_MARPT_Cont_49**	321	52.0	5,133	307	658	15,991	956	2,050	0.02	0.001	0.002	16.7	7.8	1	3
**11**	**Lsat_MARPT_Cont_54**	217	32.7	36,184	3,347	9,905	166,747	15,424	45,645	0.13	0.01	0.04	10.8	3.7	36	166
**12**	**Lsat_MARPT_Cont_61**	238	38.2	27,179	5,034	6,901	114,197	21,151	28,996	0.10	0.02	0.02	5.4	3.9	23	97
**13**	**Lsat_MARPT_Cont_65**	260	37.7	45,522	10,213	18,782	175,085	39,281	72,238	0.16	0.04	0.07	4.5	2.4	40	154
**14**	**Lsat_MARPT_Cont_70**	185	45.4	10,256	1,094	2,338	55,438	5,914	12,638	0.04	0.00	0.01	9.4	4.4	19	103
**15**	**Lsat_MARPT_Cont_74**	217	33.6	21,219	2,212	2,480	97,783	10,194	11,429	0.08	0.01	0.01	9.6	8.6	24	111
**16**	**Lsat_MARPT_Cont_82**	197	25.4	12,971	5,597	7,227	65,843	28,411	36,685	0.05	0.02	0.03	2.3	1.8	24	122
**17**	**Lsat_MARPT_Cont_83**	181	32.6	10,414	1,184	1,482	57,536	6,541	8,188	0.04	0.004	0.01	8.8	7.0	17	94
**18**	**Lsat_MARPT_Cont_91**	255	39.6	17,399	3,380	4,904	68,231	13,255	19,231	0.06	0.01	0.02	5.1	3.5	22	86
**19**	**Lsat_MARPT_Cont_94**	240	43.8	43,818	2,329	8,788	182,575	9,704	36,617	0.16	0.01	0.03	18.8	5.0	37	154
**20**	**Lsat_MARPT_Cont_95**	188	38.3	13,236	2,808	3,342	70,404	14,936	17,777	0.05	0.01	0.01	4.7	4.0	15	80
**21**	**Lsat_MARPT_Cont_113**	223	41.3	12,213	3,110	4,310	54,767	13,946	19,327	0.04	0.01	0.02	3.9	2.8	30	135
**22**	**Lsat_MARPT_Cont_124**	211	49.3	2,141	215	373	10,147	1,019	1,768	0.01	0.001	0.001	10.0	5.7	1	5
**23**	**Lsat_MARPT_Cont_138**	198	41.4	17,234	3,850	5,503	87,040	19,444	27,793	0.06	0.01	0.02	4.5	3.1	18	91
**24**	**Lsat_MARPT_Cont_142**	235	41.3	26,304	3,402	5,911	111,932	14,477	25,153	0.09	0.01	0.02	7.7	4.5	20	85
**25**	**Lsat_MARPT_Cont_144**	362	47.5	6,408	619	1,014	17,702	1,710	2,801	0.02	0.002	0.004	10.4	6.3	1	3
	**TOTAL**			1,789,178	312,721	504,052				6.39	1.12	1.80	5.72	3.55		

Number of mapped reads from control and normalized genomic libraries to simple repeats (1–3), centromeric (4), ribosomal RNA (5–6), retrotransposon *pol* consensus (7), and other uncharacterized genomic repeats (8–25). Twenty eight million reads were analyzed from each library.

#### Ribosomal genes

Sequences corresponding to 18S and 28S rRNAs were also dramatically reduced in the normalized genomic libraries (Table 2). Reduction of both of these sequences, especially the 28S rRNA, was more efficient in the library renatured with 0.5 M NaCl, even though the average GC content of 28S RNA genes is 57.9%.

#### Transposable element sequences and additional, undefined repeats

A large fraction of the lettuce genome is composed of a variety of transposable elements, particularly retrotransposons (The Lettuce Genome Consortium, unpublished). We therefore analyzed the effect of normalization on *pol* gene sequences of a long terminal repeat (LTR) retrotransposon. We generated a *pol* consensus sequence by alignment and extension of Illumina reads from our control genomic library (see Materials and Methods). The resulting lettuce *pol* consensus sequence was 5 kb long and had an uninterrupted ORF encoding a protein of 1255 amino acids belonging to *Ty1*/*Copia* retrotransposon family. Alignment of reads from the control genomic library with at least 80% identity to the *pol* consensus sequence indicated that 2% of the lettuce genome consists of complete or partial *pol-*related sequences. In the DSN-treated libraries, *pol*-related sequences (mapped at >80% s identity) were reduced approximately 3 to 5 times when renaturation was carried out in 3 M TMAC and 0.5 M NaCl respectively (Table 2). The amount of depletion/reduction of eighteen additional genomic repeats varied from 2.3 to 18 fold depending on their abundance and GC content as well as their sequence complexity and heterogeneity (Table 2). GC content was again a major factor influencing the extent of depletion and the difference between the NaCl and TMAC treatments was greater for sequences with higher GC content. Repeat reduction tended to be inversely correlated with sequence divergence as expected based on the kinetics of hybridization.

### Analysis of Repeated Sequences and Single-copy Genes Contained in a BAC Clone

**Figure 7:**
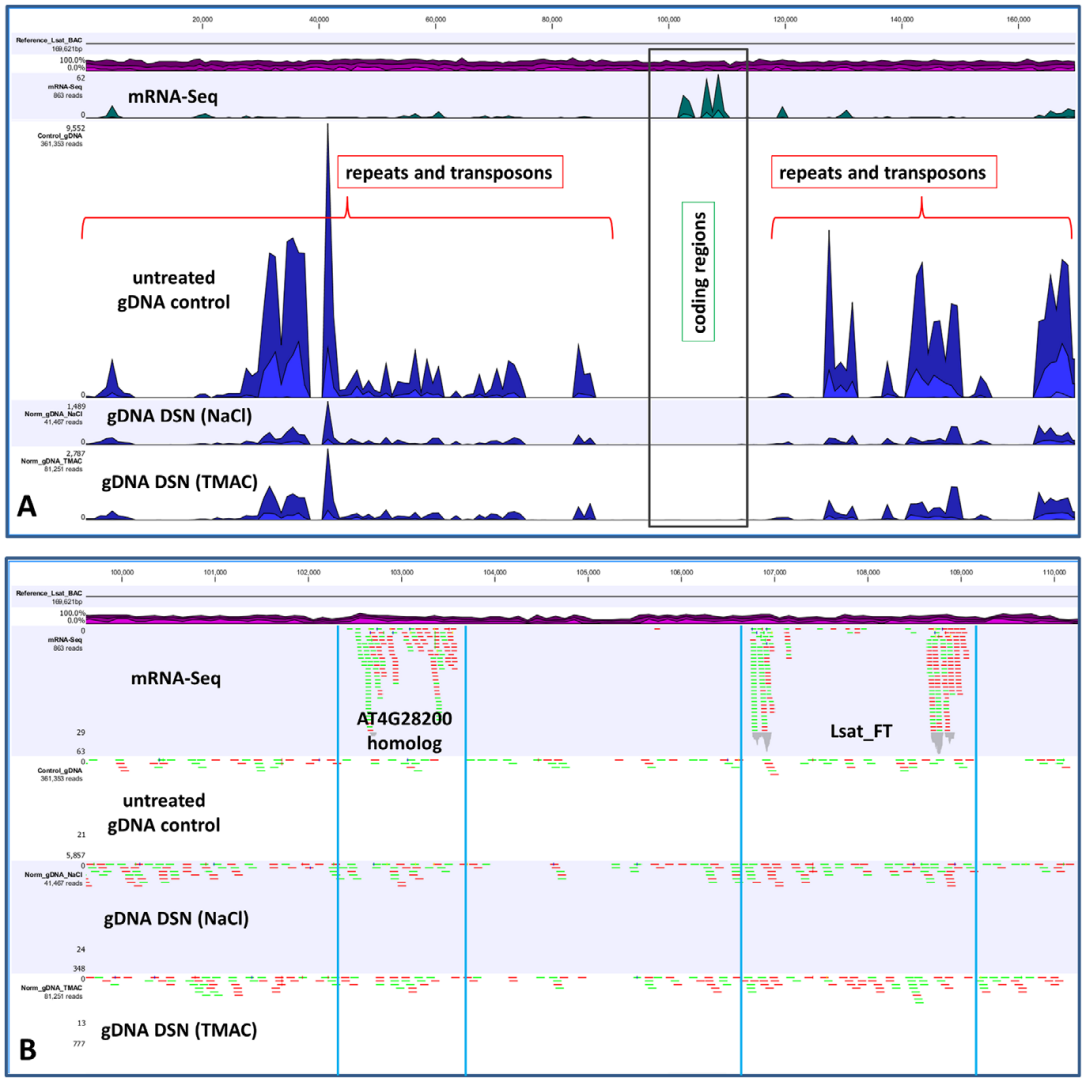
Reduction of repeated sequences and increases of single-copy gene sequences encoded in a BAC clone in normalized genomic libraries. A) The abundance of reads from four lettuce libraries: RNA-Seq (RNA-Seq), non-normalized genomic (untreated gDNA control), genomic normalized after hybridization in 0.5 M NaCl (gDNA DSN (NaCl)) and genomic normalized after hybridization in 3 M TMAC (gDNA DSN (TMAC)). The track above the RNA-Seq data shows the GC content. B) The region of A) enlarged to reveal the numbers of sequencing reads that mapped to two single-copy genes in the BAC. The homolog of At4g28200 is contained between the two vertical blue lines on the left side of the figure and the *FT* homolog is contained between the two vertical blue lines on the right side of the figure.

The complete sequence of a BAC clone was used to visualize the depletion of repeated sequences and enrichment of single-copy gene sequences across an individual region of the lettuce genome. A BAC clone (GenBank accession number JX485630) had previously been identified as encoding a homolog of the Arabidopsis *FLOWERING LOCUS T* gene (*FT*; At1g65480) and its sequence determined by Sanger sequencing (D. Lavelle and R. Michelmore, unpublished). Twenty-eight million Illumina sequencing reads from each of three genomic libraries (one control and two normalized: one after hybridization in NaCl, the other in TMAC) were mapped to the entire BAC sequence. Many more reads in the control genomic library corresponded to the repetitive sequences in this BAC clone than did reads in the normalized libraries (Figure 7A). Numbers of mapped reads from normalized libraries were 8 to 5 fold less than in the control library. RNA-Seq reads were mapped to two genic regions in the BAC sequence, the *FT* homolog and a homolog of At4g28200, a gene that may be involved in RNA processing. The numbers of reads corresponding to these two genes were increased in both normalized genomic libraries by 1.4 to 2 and 2 to 3 fold for the *FT* and At4g28200 homologs respectively (Figure 7B).

### Effect of Normalization on Protein-coding Sequences in Genomic Libraries of Lettuce

**Figure 8:**
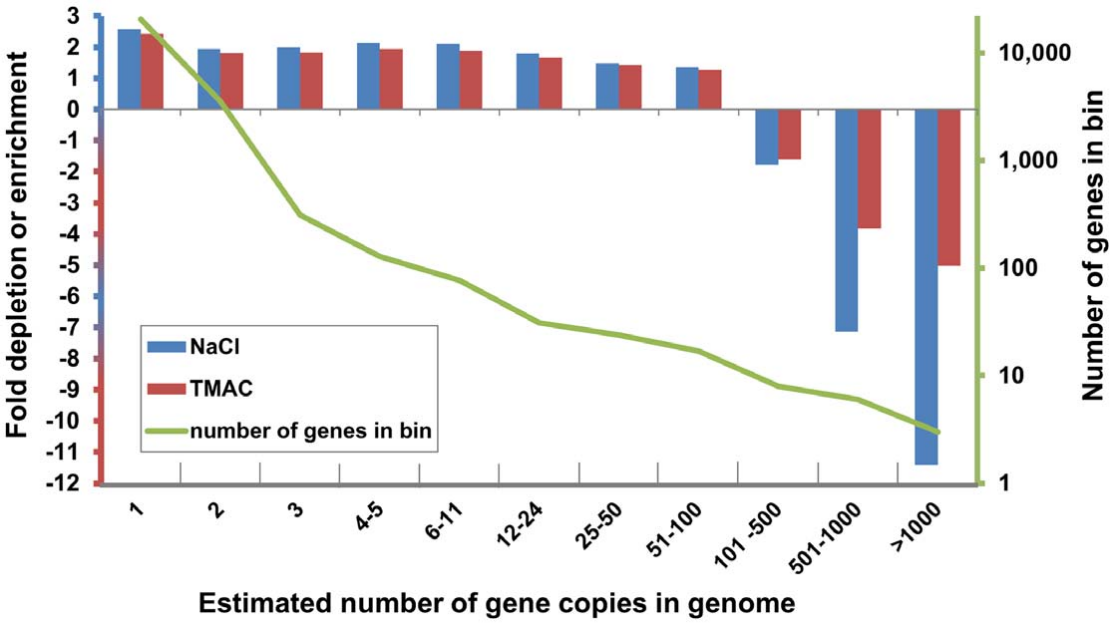
Depletion or enrichment of gene sequences in genomic libraries is dependent on the number of copies of the gene in the genome. Sixty million reads from each library, normalized using 0.5 M NaCl (blue bars) or 3 M TMAC (red bars), were mapped to a set of 25,857 lettuce transcriptome contigs with uninterrupted ORFs; the contig/gene sequences were then placed in bins based on estimates (see text) of the number of copies of each gene in the lettuce genome. A transition from enrichment of gene sequences present in the genome in fewer copies to depletion of sequences present in more than 100 copies was observed.

We investigated the effect of DSN normalization on the most abundant gene families in lettuce. The annotation derived from a BLAST search was compared to Arabidopsis-predicted proteins (http://www.arabidopsis.org/) and independently to a Pfam HMM search ([50]; http://hmmer.janelia.org/) for protein domains (see Materials and Methods). We mapped 60 million reads from each library to a set of 25,857 transcriptome contigs with uninterrupted ORFs. A subset of 1,587 single-copy genes was used to estimate coverage of unique genomic elements; the control library averaged 18 reads per single-copy gene. We used this estimate of reads/gene copy to place all of the coding gene sequences in bins based on their copy numbers. A transition from enrichment to depletion was observed (Figure 8, Table S3). The single-copy genes were enriched by up to 2.45 fold in DSN-treated samples. Genes present at up to 46 copies were also enriched. There was no depletion detected for multi-gene families that had up to ca.100 copies. In contrast, multi-gene families present in ca. 1000 copies were depleted by 7.6 and 4 times when hybridized in 0.5 M NaCl, and 3 M TMAC respectively. Most of these highly abundant sequences were transcribed from transposons or retrotransposons.

We then specifically examined the effect of DSN normalization on sequences identified in the Pfam HMM search that encode various common protein domains including kinases, transferases, cytochrome p450 and the leucine-rich repeat (LRR) domain associated with disease resistance genes (Table 3). In all cases, the protein-coding sequences were enriched in the normalized libraries by more than two fold despite being part of large multigene families. The lack of reduction reflects the sequence divergence between paralogs. While average enrichment of most of the 11 sequences examined was slightly higher in the library renatured in 0.5 M NaCl compared to the library renatured in 3 M TMAC, two transferase sequences with relatively high GC content were more enriched in the library normalized in 3 M TMAC (Table 3). A peroxidase sequence and an RNA recognition motif with similarly high GC contents, however, were somewhat more enriched in the library normalized in 0.5 M NaCl.

**Table 3:** The effect of DSN normalization on the prevalence of the sequences containing the twelve most abundant Pfam domains.

Description	Pfam_ID	Number of contigs with detected Pfam domain in transcriptome assembly	Average GC%	Average # of reads per Kb CONTROL	Average# ofreads perKb NaCl	Average# ofreads perKb TMAC	Enrichment Control/NaCl	Enrichment Control/TMAC	RatioNaCl/TMAC	contig with max number of reads in CONTROL	Max NaCl	Max TMAC
Protein kinase domain	Pkinase	938	42.5	21.3	51.6	48.6	2.42	2.28	1.06	131.5	238.5	186.5
PPR (pentatricopeptide) repeat	PPR	364	38.9	27.7	69.3	60.2	2.50	2.17	1.15	108.6	227.3	247.5
Cytochrome P450	p450	241	43.1	25.9	62.3	59.3	2.41	2.29	1.05	165.6	342.2	283.8
WD domain, G-betarepeat	WD40	136	43.1	20.1	45.1	41.4	2.24	2.06	1.09	97.8	163.2	163.2
NB-ARC domain	NB-ARC	116	39.6	33.3	84.2	70.8	2.53	2.13	1.19	394.3	965.1	809.1
Leucine Rich Repeat	LRR_1	108	42.4	23.5	66.9	63.8	2.85	2.71	1.05	53.7	118.4	86.7
Myb-like DNA-binding domain	Myb_DNA-binding	98	43.3	21.2	47.3	43.0	2.23	2.03	1.10	36.5	86.2	73.0
RNA recognition motif	RRM_1	98	44.4	17.2	38.7	34.4	2.25	2.00	1.13	40.4	110.9	90.7
UDP-glucoronosyl and UDP-glucosyl transferase	UDPGT	83	45.2	24.0	55.4	58.1	2.31	2.42	0.95	53.3	56.7	90.0
TIR domain	TIR	74	40.2	28.6	65.3	57.5	2.28	2.01	1.14	112.1	296.0	275.9
Transferase family	Transferase	73	44.2	23.2	54.6	57.3	2.35	2.47	0.95	51.9	84.7	71.0
Peroxidase	peroxidase	69	44.6	24.3	54.2	53.6	2.23	2.21	1.01	124.2	247.3	290.9

## Discussion

The DSN protocol provides efficient access to the low-copy fraction of genomes and rare components of transcriptomes. It decreases the numbers of reads and hence the cost required for sequencing as well as increases the ease of assembly of the most informative components of a genome. It also reduces contamination from chloroplast and mitochondrial sequences to acceptable levels compatible with the throughput of next-generation sequencers. It can be applied to *de novo* sequencing or re-sequencing of any organism with a large genome. It allowed us to generate comprehensive transcriptome and gene-space assemblies of lettuce. Our protocol has now been successfully applied to diverse species including *Bremia lactucae* (J. Wong & R. Michelmore, unpublished), *A. arenosa* (J. Monson-Miller & L. Comai, unpublished), cotton (M. Matvienko & A. Van Deynze, unpublished), and *Triphysaria spp.* (M. Matvienko & J. Yoder, unpublished).

Libraries for Illumina sequencing are ideally suited to reducing highly repeated sequences by exploiting renaturation kinetics. The short 300 to 500 bp fragments allow reduction of repeated sequences without concomitant loss of neighboring low-copy sequences. DSN normalization has been used widely to normalize transcriptomic libraries [51] and use of the method on RNA-Seq libraries prepared for Illumina sequencing has recently been reported [52]. However, detailed analysis of the consequences of the DSN treatment for transcriptomic libraries had not been made. The most highly abundant sequences in the control libraries were the most effectively reduced in the normalized libraries. Therefore, the normalized libraries had greater representation of rare transcripts and were suitable for gene discovery. The most abundant sequences in the control libraries still tended to be the most abundant in the normalized libraries. Therefore, it may be possible to make qualitative inferences about differences in gene expression that could be validated by gene-specific approaches such as qPCR. To avoid errors due to differential digestion when making comparisons between samples (such as in RNA-Seq), samples should be indexed and pooled prior to treatment. The amount of depletion that we observed varied among similarly prevalent sequences and was influenced by GC content and copy number in addition to sequence identity. It is, therefore, not possible to make general inferences as to the threshold of sequence identity required for this protocol to be effective. Nevertheless, we observed enrichment rather than reduction of divergent MADS-box transcription factor family, AP2/EREBP transcription factor family, and ABC superfamily members in repeat-reduced libraries; this indicates that hybridization between non-identical gene family members is infrequent and does not result in their digestion. Renaturation in 3 M TMAC rather than 0.5 M NaCl partially but not fully compensated for GC content. The DSN approach has recently been extended to RNA-Seq using total RNA as the starting material because it has the potential to reduce the prevalence of ribosomal RNA sequences (http://www.illumina.com/applications.ilmn); however, this protocol has not previously included TMAC to ameriolate the GC bias.

Under the conditions of our experiments for construction of gene-space libraries, the prevalence of highly repeated sequences was greatly reduced but not eliminated. There was progressively less reduction as the copy number of the sequence decreased. Sequences with up to 43 copies in lettuce, however, were enriched rather than depleted in the normalized libraries. Consequently, it should be possible to detect copy-number variation of such genes in normalized genomic libraries.

Given the on-going decreases in sequencing costs, DSN treatment becomes less valuable for species such as Arabidopsis with fairly simple genomes because only a small proportion of their genomes are repeated and therefore depleted by DSN treatment. In species with genomes of approximately 1 Gb or less the benefits of DSN treatment are probably marginal. For species with genomes of more than 2 Gb, there is the potential for significant benefit that increases with increasing genome size. We observed an overall reduction of the 2.7 Gb lettuce genome, comprised of over 70% repeated sequences, to 1.16 Gb of gene-space. Application of DSN normalization to species with very large genomes, for example wheat (16 Gb; [53]), *Pinus* spp. (20 to 40 Gb; [54]), *Allium* spp. (26 to 80 Gb; [55]), and lily (90 Gb; [56]), may enable efficient access to the gene space of one or more genotypes of these species. Inclusion of TMAC might also increase the applicability of the DSN method for metagenomic experiments involving multiple species with varying GC contents [57].

The degree of gene-space enrichment will vary between species and depend on the size of the genome and its history of expansion and consequent sequence complexity of the repeated components. Simple sequence, low complexity repeats are depleted more efficiently than more complex repeated sequences because of the increase in the probabilities of forming DNA duplexes. Genomes that have expanded recently are likely to be depleted more than genomes that have undergone ancient expansions and therefore contain repetitive elements that have diverged to the point that they do not cross-hybridize at 68°C. Evolutionarily young repetitive elements will tend to be eliminated more effectively than more ancient elements using the DSN protocol. We observed at least a two-fold reduction in the *Athila* and *pol* retrotransposon sequences that we analyzed in Arabidopsis and lettuce respectively. The fraction of reads with telomeric repeat sequences was reduced four fold in Arabidopsis and more than 100 fold in lettuce. The centromeric regions of *A. thaliana* contain ∼15,000 copies of a 178-bp satellite repeat [58]; these were reduced by a factor of 5 in Arabidopsis and by ∼1.5 to over 2 fold in lettuce. Genes encoding ribosomal RNA (rRNA) exist in large tandem arrays and their copy numbers vary widely among eukaryotic species, from approximately 40 to >19,000 in animals and from 150 to >26,000 in plants [59]. We observed reductions in ribosomal sequences ranging from 10 to more than 30 fold in Arabidopsis and from 5 to 20 fold in lettuce. The efficient reduction of rDNA sequences is consistent with their high GC content. Extrapolating the reduction of Arabidopsis reads representing 2.4 kb of rDNA sequence to the entire 10 kb gene [60], a 20-fold reduction of the rDNA reads alone indicates a reduction of about 7.1 Mb of genomic sequence equivalent to approximately 5% of the Arabidopsis genome.

While effective, the protocol reported here is not necessarily optimal and further improvements may be possible. In particular, the effect of DSN treatment on total RNA libraries in reducing ribosomal DNA has yet to be reported for plants. Also, for genomic libraries, shorter reannealment times, higher stringency hybridization buffers, and buffer additives like betaine in place of TMAC have yet to be investigated.

This protocol will make the resequencing of large genomes markedly more efficient. As high-quality transcriptomic or genomic reference assemblies are completed for many species, emphasis is shifting to sequencing multiple individuals within a species in order to identify sequence and copy-number variants in the low-copy fractions of genomes as well as individuals in segregating populations for mapping purposes. The high-copy fraction of a genome is uninformative and obstructive for such studies. Therefore, it is desirable to quickly and efficiently sequence the coding and associated regulatory regions; DSN normalization may be particularly applicable to human genomes for the purposes of diagnostics and personalized medicine. It is considerably easier and less expensive than various exome capture approaches currently available ([19] and references therein). Resequencing the gene-space will be useful for agricultural applications as well. We have used this normalization protocol to comprehensively sample the transcriptomes of multiple lettuce genotypes, allowing efficient discovery of SNPs (L. McHale & R. Michelmore, unpublished). We are also using this approach to analyze lettuce recombinant inbred lines segregating for disease-resistance genes and other agriculturally important traits to generate ultra-dense SNP-based maps and molecular markers for crop improvement.

## Materials and Methods

### Preparation of Libraries

Genomic DNA was isolated from leaves of Arabidopsis and lettuce using a modified CTAB extraction method [61]. DNA samples were fragmented to lengths of ∼300 bp by sonication using a UCD-200 BioRuptor as per the manufacturer’s instructions (Diagenode, Inc., Denville, NJ, USA). Genomic paired-end libraries were then prepared using paired-end DNA library construction kits available from Illumina and following the manufacturer’s instructions (Illumina Inc., San Diego, CA, USA).

Total RNA was isolated from young leaves of lettuce cultivar Valmaine using the method of Pawlowski et al. [62]. The following tissues were collected and used for isolation of total RNA from lettuce cv. Salinas: seeds that had been imbibed in water and then subjected to light or dark conditions for 24 hrs; roots (excluding the tap root) from four-weeks-old plants, hydroponically grown in Hoagland media [63]; etiolated seedlings grown from seeds that had been imbibed in water at 20°C for 12 hrs in the light then transferred to dark for 69 hrs; shoot apical meristems which had been dissected from plants with 5–6 true leaves (approx. 6 weeks old); defense-induced leaves that had been harvested 24 hrs after treatment; defense-induced seedlings that had been harvested 30 mins after treatment; flower buds 2–8 mm long; flowers at anthesis and 6 days post-anthesis. Defense-induction (to ensure that defense-related genes were expressed) comprised treatment of leaves with 40 mM salicylic acid or 1.2 mM benzothiadiazole and seven-day-old seedlings with flg22 peptide [64] in 10 mM Hewitt nutrient solution [65]. All tissues were collected directly into liquid nitrogen and stored at −80°C until used for RNA isolations. The protocol used by Cooley et al. [66] was followed for isolations of total RNA from lettuce seeds; total RNA from all other tissues of the Salinas cultivar was isolated using RNeasy Maxi kits as described in the Qiagen protocol. Approximately equal amounts of total RNA isolated from each Salinas tissue were then pooled prior to poly(A) RNA preparation. All polyA RNA was isolated using Dynal oligo-dT beads and following the manufacturer’s procedure (Invitrogen Inc., Carlsbad, CA, USA). mRNA libraries were prepared using RNA-Seq kits as per the manufacturer’s instructions (Illumina Inc., San Diego, CA, USA).

### Procedure for Normalization of Genomic and RNA-Seq Libraries

The libraries were denatured at 98°C, renatured at 68°C, and treated with DSN followed by re-amplification of the undigested fragments. Renaturations in initial studies were carried out in 0.5 M NaCl. For these experiments, up to 3 µl of a 4 µl reaction volume consisted of the nucleic acid sample. One µl of a 4× NaCl renaturation buffer comprised of 200 mM HEPES, pH 7.5 and 2 M NaCl was then added to complete the 0.5 M NaCl samples. The final protocol for normalizing most of the transcriptomic and genomic libraries involved reannealing the denatured nucleic acids in buffer containing 3 M TMAC and 200 mM HEPES, pH 7.5 at 68°C for 5 or 22 hours, respectively, followed by DSN treatment and library amplification. The detailed protocol is provided in Text S1. Renaturation, DSN treatment and library amplification were carried out as described for the final protocol except as noted in the text.

### Illumina Sequencing and Filtering for High Quality Reads

All sequencing was performed using Illumina Genome Analyzer II and HiSeq 2000 instruments according to the manufacturer’s specifications. High quality sequences were extracted from the primary output of the Illumina sequence analysis pipeline (qseq files) prior to assembly. Quality scores in qseq files were parsed (http://code.google.com/p/atgc-illumina/) and everything after the first failed score (‘B’) was trimmed (see http://code.google.com/p/atgc-illumina/wiki/Illumina_Quality_Scores).

### Transcriptome Assembly

After trimming, approximately 80 million sequences from the *Lactuca sativa* RNA-Seq libraries, ranging between 40 to 80 nt in length and between 20 to 80% in GC content, were selected for the transcriptome assembly. High quality reads were assembled using Velvet [46] with incremental K-mer values (33 to 55 with step 2) and CLC Genomic Workbench (http://clcbio.com/index.php?id=1240). Contigs generated using Velvet and CLC were combined into a mega-assembly using CAP3 [47]. The final assembly was validated by re-aligning the raw reads to the consensus sequences and then submitted to the NCBI GenBank Transcriptome Shotgun Assembly Sequence Database under accession JI573761 to JI625602. Details of the assembly procedure are publicly available at the Google Code web site: http://atgc-illumina.googlecode.com/files/PAG_2010_AKozik_V09.pdf.

### Gene-space Assembly

After trimming, approximately 450 million sequences from paired-end genomic libraries, ranging in length between 40 and 125 nt, were used for *de novo* assembly of the gene space of *L. sativa* using CLC Genomics Workbench 4.5.1 (Table S4). The resulting 876,110 lettuce contigs were submitted to GenBank: http://www.ncbi.nlm.nih.gov/Traces/wgs/?val=AFSA01.

### Lettuce Transcriptome QC Set

#### Extraction of CDS regions with uninterrupted ORFs in lettuce transcriptome assembly

Transcriptome contigs (51,842) were aligned with the NCBI plant protein reference set (plant RefSeq; [67]) using a BLAST (blastx) search with expectation value threshold of 1e^−20^. Regions of contigs aligned to plant RefSeq sequences were then extracted using custom scripts (http://cgpdb.ucdavis.edu/SNP_Discovery_CDS/). Sequences with internal stop codons were discarded. The resulting set of CDS transcriptome fragments was blasted against itself to identify and remove possible duplicates. This procedure generated 25,857 non-reduntant, putative ORFs with high degrees of sequence similarity to known proteins (Table S5).

#### Identification of single-copy genes

Single-copy genes ( = a subset of 25,857 transcriptome QC set) were identified by BLAST search of CDS transcriptome regions versus themselves to select sequences with only a strong single hit. Additional filtering included selection for complete ORFs based on presense of an ATG start codon and putative protein length (based on alignment to known protein sequences). Additionally, candidates were BLASTed versus our lettuce gene-space assembly and sequences with multiple hits were filtered out. Upon successfully passing these filtering critera, 1,587 transcriptome contigs were identified as putative single-copy genes with complete uninterrupted ORFs in the lettuce genome.

### Lettuce Repeat Sequences

#### Pol consensus and additional, uncharacterized repeats

Ten thousand of the most abundant 30-mers extracted from 10 million were analyzed using our SeqExter program (http://code.google.com/p/atgc-wint/). SeqExter builds a consensus sequence based on seeded alignments by extension. A consensus sequence was generated for each 30-mer seeded alignment. Consensus sequences were further assembled using CAP3 [47]. All the resulting contigs were BLASTed versus each other to identify closely related sequences. This resulted in 17 distinct highly abundant genomic repeat sequences of 180 to 360 nt.

A consensus sequence for the *pol* sequences of lettuce LTR retrotransposons was also generated using SeqExter. Original seeds for the *pol* consensus sequence were identified by BLASTing the *pol* sequence from Arabidopsis (RVT_2 Pfam family PF07727) versus lettuce genomic raw Illumina reads. The resulting consensus was checked for ORFs and conserved domains using NCBI BLAST. Presence and order of protein domains related to plant retrotransposons indicated that the identified lettuce *pol* consensus sequence belongs to the *Ty1*/*Copia* family. In addition, one longer uncharacterized consensus sequence, Lsat_XCons_B449_2012_05 (5,888 nt), was similarly built using a sequential extension approach. (See Text S2 for the lettuce *pol* consensus sequence and other uncharacterized repeat sequences used in this study.).

#### BAC

In a previous study, a BAC library of *Lactuca sativa* cv. Diana [68] was screened for a lettuce ortholog of the Arabidopsis *FLOWERING LOCUS T* (*FT*) gene At1g65480 (D. Lavelle and R. Michelmore, unpublished); Sanger sequencing of a shotgun library of the BAC clone yielded ∼6× coverage; PCR followed by Sanger sequencing was used to fill gaps between contigs to produce the complete 169,621 bps-long genomic fragment (GenBank accession number JX485630). Coding regions of the BAC clone were identified by mapping of RNA-Seq Illumina reads to the BAC sequence. Regions containing repeated elements and transposons were identified by mapping genomic reads to the BAC sequence and conducting a BLAST search versus the non-redundant GenBank protein database (http://blast.ncbi.nlm.nih.gov/Blast.cgi).

### Mapping Reads to Reference Sequences

Reads for Arabidopsis genomic libraries were mapped, by using BLASTN, to the reference sequences versus Illumina datasets. BLAST parameters (expectation values) were adjusted so that the shotest reads (24 nt) in alignments had 100% identity to reference sequences.

Analyses of lettuce RNA-Seq and genomic libaries were carried out using CLC Genomics Workbench (www.clcbio.com). Mapping parameters were 0.75 sequence overlap in all experiments and the following sequence identities: 98% - stringent; 80% - weak (as indicated in the text and tables). Values for numbers of mapped reads and coverage were extracted from CLC Genomics Workbench reports.

Analysis and visualization of reads mapping to the BAC sequence were carried out using the CLC-track tools.

## Supporting Information

Figure S1 **The majority of coding regions in Arabidopsis are not reduced as a result of DSN treatment.** Boxplots of 25 bins, based on RPKM values, showing distribution, IQR, outliers (black dot) and statistical significance (diamond in IQR). The whiskers encompass 1.5 of the interquartile range (IQR). The confidence diamonds indicate the average RPKM fold change between the control genomic library and the library normalized with DSN after 22 hrs of renaturation when Student t-test p-value is less than 0.01.
(TIF)

Figure S2 **The effect of GC content on the normalization of Arabidiopsis mitochondrial sequences.** Renaturation was carried out in 0.5 M NaCl for 22 (yellow) and 70 (red) hours prior to DSN treament. The 366,924 nt of the Arabidopsis mitochondrial genome [69] were divided into a contiguous tiling path of 500 nt fragments with 10 nt overlap and the GC content of each fragment determined. The number of reads from each fragment was determined using BLAST and the ratio of reads in the control versus the repeat-reduced libraries (# reads control/# reads DSN-treated) was plotted versus their GC content. Exponential curves that fit the data are indicated for each time point. The higher the GC content of each fragment, the more it was reduced by normalization.
(TIF)

Figure S3 **Alignment of intron positions in a lettuce gene-space assembly with the corresponding Arabidopsis gene.** Lettuce transcriptome contigs were aligned using BLASTN to the gene-space assembly revealing the intron (red lines)-exon (blue rectangles) structure. In this example, the positions of the 13 introns in the lettuce gene model homologous to the ubiquitin protein ligase gene At4G221070 exactly matched the positions of the introns (green lines) in the corresponding gene in Arabidopsis.
(TIF)

Table S1 **The effect of DSN treatment on gene prevalence in RNA-Seq libraries of lettuce cv.** Valmaine for genes expressed at different levels.
(DOCX)

Table S2 **Hybridization in 3 M TMAC reduced GC bias associated with renaturation of Arabidiopsis chloroplast sequences: statistical analysis.**
(DOCX)

Table S3 **Depletion or enrichment of gene sequences in genomic libraries is dependent on the number of copies of the gene in the genome.**
(DOCX)

Table S4 **Libraries used for the assembly of the gene-space of lettuce.**
(DOCX)

Table S5 **Non-redundant, putative ORFs of lettuce with high degrees of sequence similarity to known proteins.**
(XLS)

Text S1 **Protocol for normalization of Illumina RNA-Seq and genomic libraries.**
(DOCX)

Text S2 **Lettuce genomic repeats used for validation of DSN normalization protocol.**
(TXT)
