# Supplementary figures and images for: Consequences of Normalizing Transcriptomic and Genomic Libraries of Plant Genomes Using a Duplex-Specific Nuclease and Tetramethylammonium Chloride
Source: PLoS One. 2013 Feb 8;8(2):e55913. doi: 10.1371/journal.pone.0055913 (PMC3568094; doi:10.1371/journal.pone.0055913)

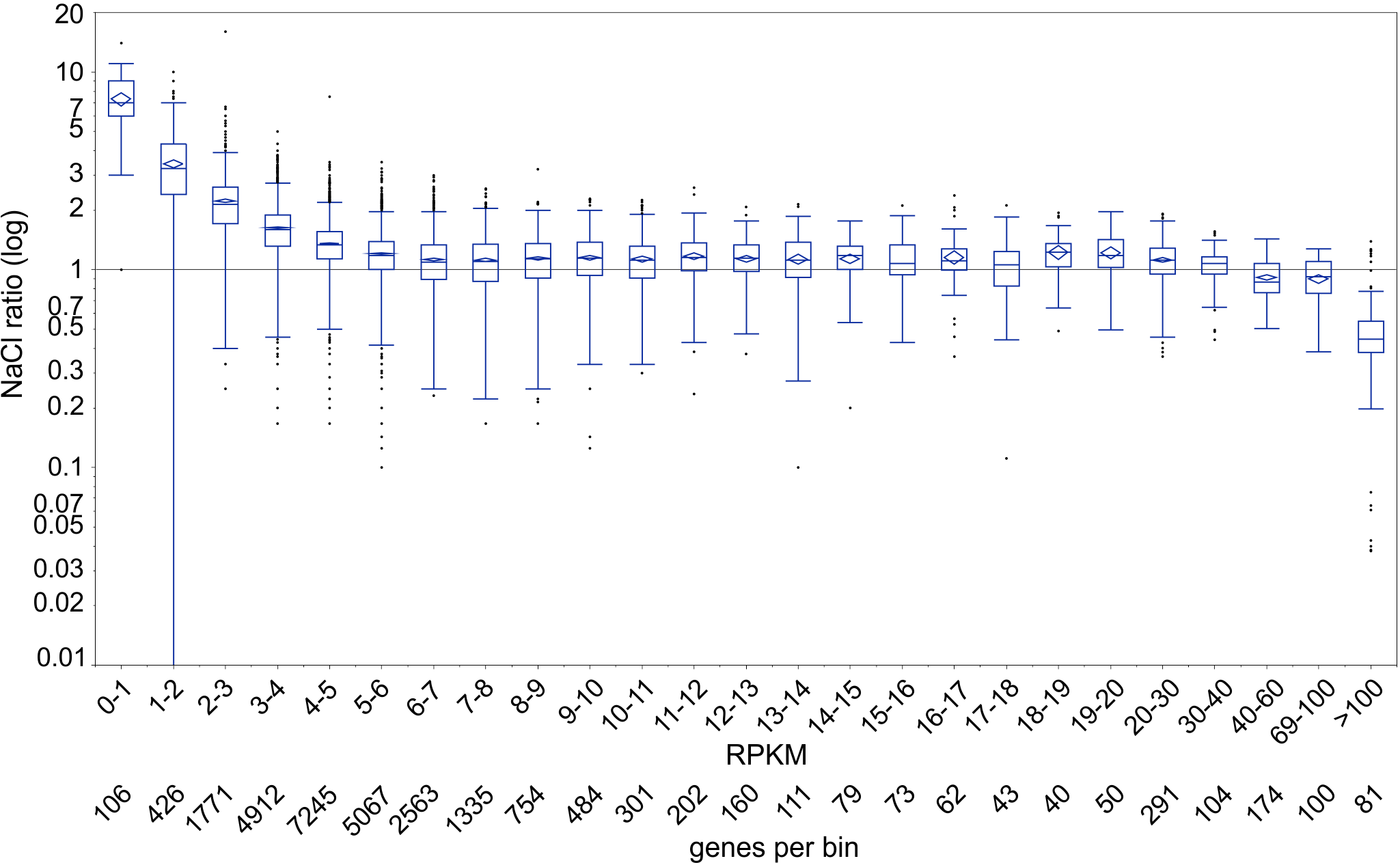

Supplement: Figure S1 — The majority of coding regions in Arabidopsis are not reduced as a result of DSN treatment. Boxplots of 25 bins, based on RPKM values, showing distribution, IQR, outliers (black dot) and statistical significance (diamond in IQR). The whiskers encompass 1.5 of the interquartile range (IQR). The confidence diamonds indicate the average RPKM fold change between the control genomic library and the library normalized with DSN after 22 hrs of renaturation when Student t-test p-value is less than 0.01. (TIF) [file pone.0055913.s001.tif]

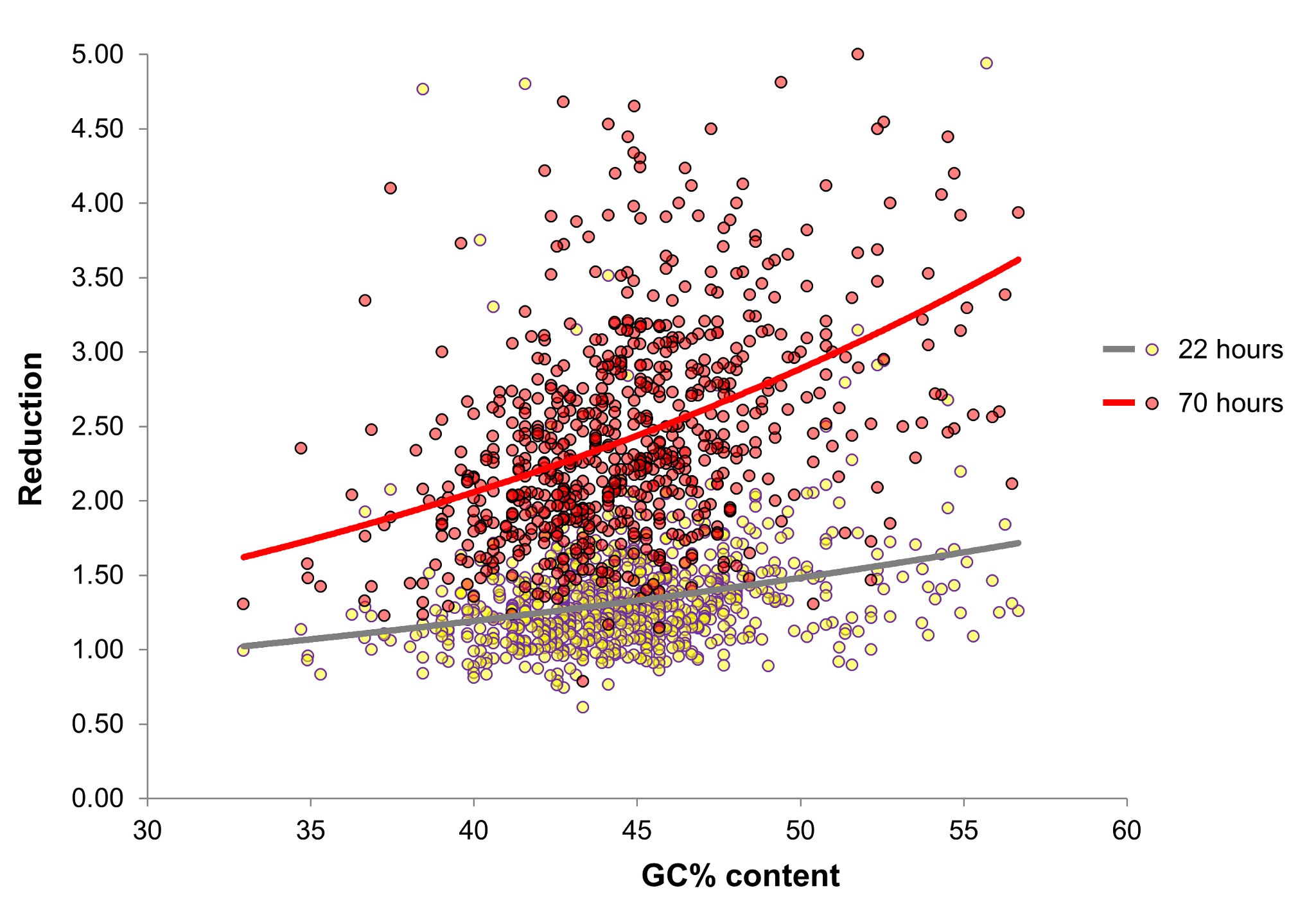

Supplement: Figure S2 — The effect of GC content on the normalization of Arabidiopsis mitochondrial sequences. Renaturation was carried out in 0.5 M NaCl for 22 (yellow) and 70 (red) hours prior to DSN treament. The 366,924 nt of the Arabidopsis mitochondrial genome [69] were divided into a contiguous tiling path of 500 nt fragments with 10 nt overlap and the GC content of each fragment determined. The number of reads from each fragment was determined using BLAST and the ratio of reads in the control versus the repeat-reduced libraries (# reads control/# reads DSN-treated) was plotted versus their GC content. Exponential curves that fit the data are indicated for each time point. The higher the GC content of each fragment, the more it was reduced by normalization. (TIF) [file pone.0055913.s002.tif]

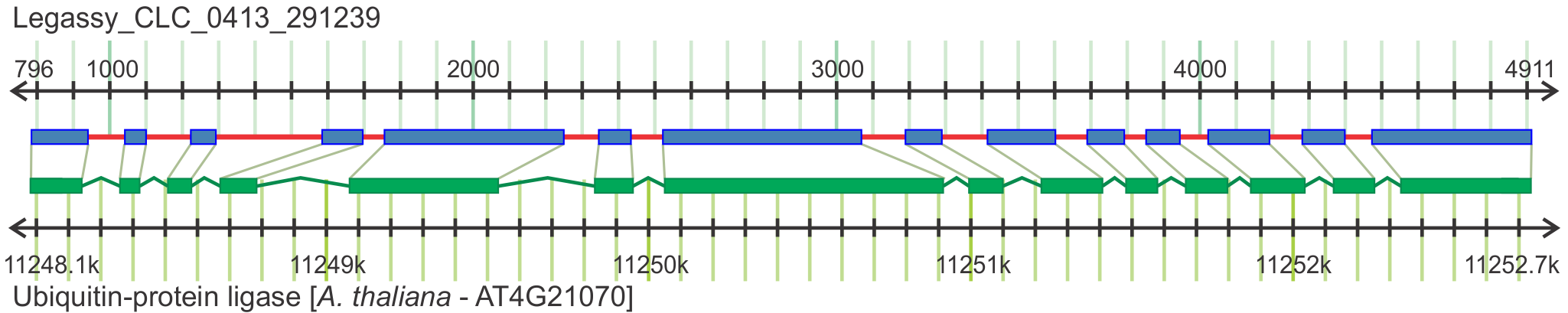

Supplement: Figure S3 — Alignment of intron positions in a lettuce gene-space assembly with the corresponding Arabidopsis gene. Lettuce transcriptome contigs were aligned using BLASTN to the gene-space assembly revealing the intron (red lines)-exon (blue rectangles) structure. In this example, the positions of the 13 introns in the lettuce gene model homologous to the ubiquitin protein ligase gene At4G221070 exactly matched the positions of the introns (green lines) in the corresponding gene in Arabidopsis. (TIF) [file pone.0055913.s003.tif]
