# Supplementary material for: Consequences of Normalizing Transcriptomic and Genomic Libraries of Plant Genomes Using a Duplex-Specific Nuclease and Tetramethylammonium Chloride
Source: PLoS One. 2013 Feb 8;8(2):e55913. doi: 10.1371/journal.pone.0055913 (PMC3568094; doi:10.1371/journal.pone.0055913)
Supplement: Table S1 — The effect of DSN treatment on gene prevalence in RNA-Seq libraries of lettuce cv. Valmaine for genes expressed at different levels. (DOCX) [file pone.0055913.s004.docx]

**Table S1.** The effect of DSN treatment on gene prevalence in RNA-Seq libraries of lettuce cv. Valmaine for genes expressed at different levels. Statistical significance of the ratio (P-values) were computed for each bin using two tails Student t-test under equal variance assumption.

| RPKM range in standard library | RPKM average in standard library | RPKM average in DSN normalized library | number of genes in bin | ratio standard/  normalized | P-value |
| --- | --- | --- | --- | --- | --- |
| 0-1 | 0.24 | 0.47 | 6277 | 1.96 | 7e-162 |
| 1-2 | 1.41 | 2.02 | 1657 | 1.43 | 5e-112 |
| 2-4 | 2.88 | 4.01 | 2076 | 1.39 | 1e-172 |
| 4-6 | 4.93 | 6.75 | 1554 | 1.37 | 1e-167 |
| 6-8 | 6.93 | 9.38 | 1365 | 1.35 | 1e-179 |
| 8-10 | 8.91 | 11.9 | 1179 | 1.34 | 1e-179 |
| 10-12 | 10.9 | 14.5 | 998 | 1.33 | 3e-159 |
| 12-14 | 12.9 | 17.0 | 927 | 1.32 | 3e-137 |
| 14-18 | 15.9 | 20.4 | 1539 | 1.28 | 4e-195 |
| 18-25 | 21.2 | 26.3 | 1971 | 1.24 | 2e-180 |
| 25-32 | 28.2 | 34.0 | 1336 | 1.20 | 7e-103 |
| 32-40 | 35.8 | 41.7 | 1012 | 1.16 | 2e-58 |
| 40-50 | 44.6 | 51.1 | 883 | 1.15 | 2e-42 |
| 50-65 | 56.9 | 62.9 | 798 | 1.10 | 1e-21 |
| 65-85 | 74.4 | 77.7 | 654 | 1.04 | 1e-4 |
| 85-100 | 92.0 | 95.1 | 277 | 1.03 | 4e-2 |
| 100-120 | 109.5 | 106.3 | 297 | 0.97 | 7e-2 |
| 120-150 | 133.9 | 122.0 | 284 | 0.91 | 3e-8 |
| 150-200 | 171.1 | 149.2 | 276 | 0.87 | 4e-15 |
| 200-300 | 243.2 | 193.2 | 230 | 0.79 | 2e-28 |
| 300-500 | 374.0 | 246.9 | 126 | 0.66 | 3e-35 |
| 500-800 | 632.4 | 328.0 | 58 | 0.52 | 1e-31 |
| 800-1500 | 1068.7 | 389.4 | 59 | 0.36 | 7e-47 |
| 1500-13000 | 3449.8 | 596.6 | 24 | 0.17 | 2e-5 |
