# Supplementary material for: Consequences of Normalizing Transcriptomic and Genomic Libraries of Plant Genomes Using a Duplex-Specific Nuclease and Tetramethylammonium Chloride
Source: PLoS One. 2013 Feb 8;8(2):e55913. doi: 10.1371/journal.pone.0055913 (PMC3568094; doi:10.1371/journal.pone.0055913)
Supplement: Table S2 — Hybridization in 3 M TMAC reduced GC bias associated with renaturation of Arabidiopsis chloroplast sequences: statistical analysis. (DOCX) [file pone.0055913.s005.docx]

**Table S2.** **Hybridization in 3 M TMAC reduced GC bias associated with renaturation of Arabidiopsis chloroplast sequences.** A) Renaturation was carried out in 0.5 M NaCl for 22 (yellow) or 70 (red) hrs prior to DSN treatment. The higher the GC content of the 500 nt fragment, the more it was reduced by DSN treatment. B) Renaturation was carried out for 22 hours in either 3 M TMAC (purple) or 0.5 M NaCl (yellow). Hybridization in 3 M TMAC reduced GC bias associated with renaturation followed by DSN treatment. Exponential curves that fit the data are indicated for each experiment. Statistical significances were assessed by two tail Student t-tests for each 5% GC content bin; fold changes (FC) and Student t-test P-value (P-val) are reported for each 5% GC content bin.

| **GC% Bin** | **Genes in Bin** | **22H FC** | **22H**  **P-val** | **70H FC** | **70H**  **P-val** | **NaCl FC** | **NaCl**  **P-val** | **TMAC FC** | **TMAC**  **P-val** |
| --- | --- | --- | --- | --- | --- | --- | --- | --- | --- |
| **18-25** | **14** | 2.43 | 2.88E-06 | 3.7 | 3.4E-08 | 0.61 | 1.9E-03 | 0.3 | 5.6E-08 |
| **25-30** | **47** | 2.67 | 9.67E-35 | 4.2 | 4.1E-43 | 1.81 | 1.3E-18 | 3.9 | 4.2E-37 |
| **30-35** | **77** | 3.42 | 9.23E-38 | 5.9 | 1.4E-45 | 2.26 | 5.7E-25 | 4.4 | 3.2E-38 |
| **35-40** | **93** | 5.13 | 5.81E-51 | 9.8 | 1.4E-57 | 3.43 | 5.4E-42 | 6.2 | 7.7E-51 |
| **40-45** | **47** | 6.37 | 8.54E-30 | 12.9 | 1.1E-32 | 4.76 | 2.1E-27 | 7.5 | 8.1E-30 |
| **45-50** | **9** | 11.4 | 5.39E-11 | 23.7 | 2.6E-11 | 11.0 | 1.9E-10 | 10.9 | 3.0E-10 |
| **50-55** | **12** | 14.3 | 1.01E-25 | 34.2 | 3.1E-26 | 19.9 | 1.3E-22 | 13.6 | 8.4E-22 |
| **55-60** | **10** | 14.9 | 6.90E-22 | 37.9 | 2.9E-22 | 23.8 | 1.1E-20 | 14.6 | 3.6E-19 |
