# Supplementary material for: Consequences of Normalizing Transcriptomic and Genomic Libraries of Plant Genomes Using a Duplex-Specific Nuclease and Tetramethylammonium Chloride
Source: PLoS One. 2013 Feb 8;8(2):e55913. doi: 10.1371/journal.pone.0055913 (PMC3568094; doi:10.1371/journal.pone.0055913)
Supplement: Table S3 — Depletion or enrichment of gene sequences in genomic libraries is dependent on the number of copies of the gene in the genome. (DOCX) [file pone.0055913.s006.docx]

**Table S3.** **Depletion or enrichment of gene sequences in genomic libraries dependent on the number of copies of the gene in the genome.** Sixty million reads from each library, normalized using 0.5 M NaCl (blue bars) or 3 M TMAC (red bars), were mapped to a set of 25,857 lettuce transcriptome contigs with uninterrupted ORFs; the contig/gene sequences were then placed in bins based on estimates (see text) of the number of copies of each gene in the lettuce genome. Fold changes (FC) and Student t-test P-value (P-val; green background when < 0.01) are reported for the 11 estimated gene copy bins.

| **number of gene copies** | 1 | 2 | 3 | 4-5 | 6-11 | 12-24 | 25-50 | 51-100 | 101 -500 | 501-1000 | >1000 |
| --- | --- | --- | --- | --- | --- | --- | --- | --- | --- | --- | --- |
| **NaCl**  **Fold Change** | 2.58 | 1.95 | 1.99 | 2.12 | 2.12 | 1.79 | 1.48 | 1.35 | -1.78 | -7.14 | -11.41 |
| **NaCl**  **P-values** | 0 | 0 | 5.5  E-96 | 2.5  E-45 | 5.26E-27 | 2.1  E-06 | 1.3  E-04 | 1.58E-03 | 0.023 | 8.2  E-06 | 4.75  E-03 |
| **TMAC**  **Fold Change** | 2.43 | 1.81 | 1.83 | 1.95 | 1.88 | 1.67 | 1.43 | 1.27 | -1.61 | -3.82 | -5.02 |
| **TMAC**  **P-values** | 0 | 0 | 1.0  E-77 | 4.8  E-38 | 1.35E-18 | 3.8  E-06 | 4. 6  E-05 | 4.99E-03 | 0.027 | 3. 4  E-05 | 8.49  E-03 |
| **number of genes in bin** | 20637 | 3580 | 313 | 128 | 77 | 31 | 24 | 17 | 8 | 6 | 3 |
