# Supplementary material for: Consequences of Normalizing Transcriptomic and Genomic Libraries of Plant Genomes Using a Duplex-Specific Nuclease and Tetramethylammonium Chloride
Source: PLoS One. 2013 Feb 8;8(2):e55913. doi: 10.1371/journal.pone.0055913 (PMC3568094; doi:10.1371/journal.pone.0055913)
Supplement: Table S4 — Libraries used for the assembly of the gene-space of lettuce. (DOCX) [file pone.0055913.s007.docx]

**Table S4. Libraries used for the assembly of the gene-space of lettuce.**

| **library code** | **hybridization conditions** | **DSN treatment** | **insert length** | **high quality reads (million)** | **read length**  **forward** | **read length**  **reverse** | **total number of nucleotides (Mb)** |
| --- | --- | --- | --- | --- | --- | --- | --- |
| LSG2 | NaCl 22 hours | + | 300±60 | (30.1) x 2 | 40-125 | 40-41 | 8,672  complete pairs |
|  | TMAC 22 hours | + | 300±60 | (26.3) x 2 | 40-125 | 40-41 |  |
| LSG2 | NaCl 22 hours | + | 300±60 | 59.3 | 40-125 | NA | 12,779  single-end run |
|  | TMAC 22 hours | + | 300±60 | 63.7 | 40-125 | NA |  |
| LSG3 | NaCl 22 hours | + | 300±60 | (41.0) x 2 | 40-105 | 40-105 | 21,015  complete pairs |
|  | TMAC 22 hours | + | 300±60 | (66.4) x 2 | 40-105 | 40-105 |  |
| **total** |  |  |  | 450.6 |  |  | **42,466** |

*Note, in addition to listed reads, about 16 million reads from paired-end runs were treated as single because they had no matching pairs resulting in a total number of reads for the assembly of 466 million.*
