# Supplementary material for: Consequences of Normalizing Transcriptomic and Genomic Libraries of Plant Genomes Using a Duplex-Specific Nuclease and Tetramethylammonium Chloride
Source: PLoS One. 2013 Feb 8;8(2):e55913. doi: 10.1371/journal.pone.0055913 (PMC3568094; doi:10.1371/journal.pone.0055913)
Supplement: Text S1 — Protocol for normalization of Illumina RNA-Seq and genomic libraries. (DOCX) [file pone.0055913.s009.docx]

## Protocol for Normalization of Illumina RNA-Seq and Genomic Libraries

**Reagents**

- Evrogen DSN kit #EA001 (distributed by Axxora, USA)
- Nuclease-free water (Ambion #AM9937)
- 100% Glycerol (USB #16374)
- Mineral oil (Sigma-Aldrich #M3516-12X6ML)
- Illumina mRNA-Seq library prep: 200 - 300 ng in 3 µl EB (10 mM Tris-Cl, pH 8.5)
- 4x NaCl Hybridization buffer: 200 mM Hepes, pH 7.5, 2M NaCl (made from: 1M HEPES Buffer Solution – Invitrogen #15630-080, 5 M NaCl solution – Ambion AM9760G)
- 0.5 M Hepes, pH 7.5
- 5 M TMAC (SIGMA #T3411)
- DSN enzyme prepared according to the instructions from Evrogen DSN kit
- 2x DSN buffer prepared from 10X DSN Master buffer supplied in the DSN kit
- 2x Phusion™ High-Fidelity PCR Master Mix (NEB F-548L)
- Illumina PCR Primer PE 1.0/SR 1.1, part #1001783
- Illumina PCR Primer PE 2.0, part #1001784
- MinElute PCR purification Kit, Qiagen #28004

**Protocol**

*This protocol is adapted from the Evrogen Manual for TRIMMER-DIRECT cDNA Normalization Kit Cat#NK002*

*NOTE: Before starting the hybridization, make sure that 4X Hybridization buffer has been allowed to stay at room temperature for at least 15-20 min. Be sure that there is no visible pellet or precipitate in the buffer before use.*

1. For each sample to be normalized in NaCl buffer combine in a 200 µl PCR tube:

3 µl Illumina RNA-Seq library or genomic paired-end library (200 – 300 ng)

1 µl 4X Hybridization buffer

For each sample to be normalized in TMAC buffer combine in a 200 µl PCR tube:

1.2 µl RNA-Seq library or genomic paired-end library (200 - 300 ng)

0.4 µl 0.5 M Hepes, pH 7.5

2.4 µl 5 M TMAC

2. Mix contents well and spin the tube briefly in a microcentrifuge.

3. Overlay the reaction mixture with a drop of mineral oil and centrifuge the tubes at 14K

rpm for 2 min.

4. Incubate the tubes in the thermal cycler:

98C – 2 min

68C – 5 hours for RNA-Seq libraries or 22 hours for genomic libraries, then proceed immediately to DSN treatment.

*NOTE: Do not remove the samples from thermal cycler before DSN treatment. The 68C step in*

*the PCR protocol should be set to “forever.”*

5. Preheat the 2X DSN buffer at 68C.

6. Add 5 µl of the preheated DSN buffer to each tube containing hybridized cDNA, spin the

tube briefly in a microcentrifuge and return it to the thermocycler. Incubate the tubes at 68C

for 10 min.

*NOTE: Do not remove the tubes from the PCR machine except for the time necessary to add*

*preheated 2X DSN buffer.*

7. Add 1 µl of DSN enzyme and return the tubes immediately to the thermocycler. Incubate at

68C for 25 min.

*NOTE: Do not remove the tubes from the PCR machine except for the time necessary to add*

*DSN enzyme. If the tube is left at room temperature after adding DSN, non-specific*

*digestion of secondary structures formed by ssDNA may occur and decrease the normalization efficiency.*

8. Add 10 µl of 2X DSN stop solution, mix contents, spin the tubes briefly in a microcentrifuge,

and place the tubes on ice.

9. Add 20 µl nuclease-free water to each tube. Mix contents and spin the tube briefly in a

microcentrifuge.

*NOTE: The samples obtained can be stored at -20C up to two weeks and used subsequently to prepare the normalized cDNA or genomic library.*

10. Prepare the following PCR reaction:

1 µl DNA from step 9

1 µl Illumina Primer PE 1.0

1 µl Illumina Primer PE 2.0

22 µl nuclease-free water

25 µl 2x Phusion mix

11. Amplify using the following PCR protocol:

a. 30 seconds at 98C

b. 12 cycles of:

10 seconds at 98C

30 seconds at 65C

30 seconds at 72C

c. 5 min at 72C

d. Hold at 10C

12. When the cycling is completed, electrophorese 2.5 µl of each sample alongside 100 ng of

“100 bp +” size marker on a 2% agarose gel. If necessary, subject the tubes to 1 to 2 additional cycles of PCR.

13. Follow the instructions in the MinElute PCR purification Kit to purify the sample and elute

in 20 µl of EB. Measure the DNA concentration. Submit 100 µl of a 10nM dilution for sequencing.
